# Hydroxycinnamoyltransferase and CYP98 in phenolic metabolism in the rosmarinic acid-producing hornwort *Anthoceros agrestis*

**DOI:** 10.1007/s00425-022-03856-9

**Published:** 2022-03-02

**Authors:** Lucien Ernst, Julia Wohl, Elke Bauerbach, Maike Petersen

**Affiliations:** Institut für Pharmazeutische Biologie und Biotechnologie, Philipps-Universität Marburg, Robert-Koch-Str. 4, 35037 Marburg, Germany

**Keywords:** Anthocerotaceae, BAHD acyltransferases, Evolution, Hydroxycinnamic acids, Hydroxycinnamoyl ester/amide 3-hydroxylase, Model organism, Phenylpropanoid derivatives, Phylogeny

## Abstract

**Main conclusion:**

*Anthoceros agrestis* hydroxycinnamoyltransferase accepts shikimic and 3-hydroxyanthranilic acids while hydroxycinnamoylester/amide 3-hydroxylase (CYP98A147) preferred *p*-coumaroyl-(3-hydroxy)anthranilic acid compared to the shikimic acid derivative. Alternative pathways towards rosmarinic acid have to be considered.

**Abstract:**

Rosmarinic acid (RA) is a well-known ester of caffeic acid and 3,4-dihydroxyphenyllactic acid. In the search for enzymes involved in RA biosynthesis in the hornwort *Anthoceros agrestis*, the hydroxycinnamoyltransferase sequence with the highest similarity to rosmarinic acid synthase from Lamiaceae has been amplified and heterologously expressed in *Escherichia coli*. In parallel, the single cytochrome P450 sequence belonging to the CYP98 group in *Anthoceros agrestis* was isolated and expressed in *Saccharomyces cerevisiae* which did not result in protein formation. Codon optimization and co-expression with NADPH:cytochrome P450 reductase (CPR) from *Coleus blumei* resulted in the formation of active enzymes. Both, the hydroxycinnamoyltransferase and CYP98 were characterized with respect to their temperature and pH optimum as well as their substrate acceptance. The hydroxycinnamoyltransferase (AaHCT6) readily accepted *p*-coumaroyl- and caffeoyl-CoA with a slightly higher affinity towards *p*-coumaroyl-CoA. The best acceptor substrate was shikimic acid (*K*_m_ 25 µM with *p*-coumaroyl-CoA) followed by 3-hydroxyanthranilic acid (*K*_m_ 153 µM with *p*-coumaroyl-CoA). Another accepted substrate was 2,3-dihydroxybenzoic acid. Anthranilic acid and 4-hydroxyphenyllactic acid (as precursor for RA) were not used as substrates. *p*-Coumaroylesters and -amides are substrates hydroxylated by CYP98 hydroxylases. The only CYP98 sequence from *Anthoceros agrestis* is CYP98A147. The best substrates for the NADPH-dependent hydroxylation were *p*-coumaroylanthranilic and *p*-coumaroyl-3-hydroxyanthranilic acids while *p*-coumaroylshikimic and *p*-coumaroyl-4-hydroxyphenyllactic acids were poor substrates. The biosynthetic pathway towards rosmarinic acid thus still remains open and other enzyme classes as well as an earlier introduction of the 3-hydroxyl group to afford the caffeic acid substitution pattern must be taken into consideration.

**Supplementary Information:**

The online version contains supplementary material available at 10.1007/s00425-022-03856-9.

## Introduction

The model plant *Anthoceros agrestis* Paton (Anthocerotaceae) belonging to the hornworts is known for its accumulation of various hydroxycinnamic acid derivatives, such as rosmarinic acid (RA) and (hydroxy)megacerotonic acid or the alkaloid anthocerodiazonin (for structures see Suppl. Fig. S1) (Takeda et al. 1990a; Trennheuser et al. 1994) as well as numerous other hydroxycinnamic and benzoic acid derivatives (Trennheuser 1992). As such a wealth of phenolic compounds has rarely been described in mosses and liverworts, investigations into the biochemical machinery of *Anthoceros* and the encoding genes are interesting also with regard to its evolutionary development. RA is better known as a typical compound of members of the seed plant families Lamiaceae (subfamily Nepetoideae) and Boraginaceae, although its wide occurrence in other taxa of the seed plants and in ferns has been documented (Petersen et al. 2009). Its biosynthesis has first been established in a member of the Lamiaceae, namely *Coleus blumei* (syn. *Plectranthus scutellarioides*, *Solenostemon scutellarioides*) (Petersen et al. 1993), and verified in other plant species (Ma et al. 2015).

**Fig. 1:**
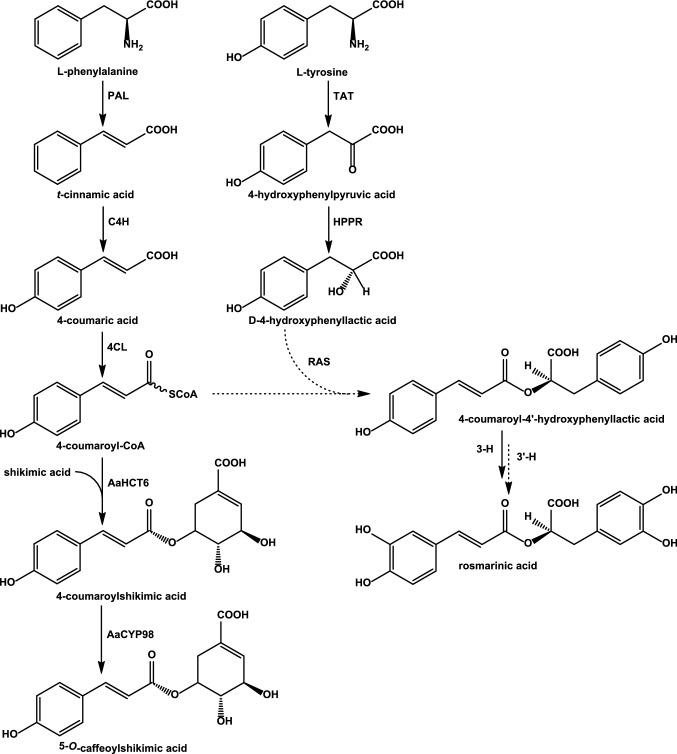
Biosynthetic pathways leading to rosmarinic acid and caffeoylshikimic acid. Abbreviations of enzyme names are as follows: *PAL* l-phenylalanine ammonia-lyase, *C4H* cinnamic acid 4-hydroxylase, *4CL* 4-coumaric acid coenzyme A ligase, *TAT* l-tyrosine aminotransferase, *HPPR* hydroxyphenylpyruvic acid reductase, *RAS* “rosmarinic acid synthase” = hydroxycinnamoyl-CoA:hydroxyphenyllactic acid hydroxycinnamoyltransferase, *3-H,* *3′-H* 3- and 3′-hydroxylase(s), *AaHCT6* 4-coumaroyl-CoA:shikimic acid hydroxycinnamoyltransferase from *Anthoceros agrestis*, *AaCYP98* 4-coumaroylshikimic acid 3-hydroxylase from *Anthoceros agrestis*. Full arrows show reactions which have been characterized in *Anthoceros agrestis*, dashed arrows reactions have not yet been characterized

The biosynthesis of the above-mentioned hydroxycinnamic acid derivatives requires the presence of the general phenylpropanoid pathway to afford activated hydroxycinnamic acids. Phenylalanine ammonia-lyase (PAL), cinnamic acid 4-hydroxylase (C4H) as well as *p*-coumarate:CoA-ligases (4CL) have previously been detected and characterized from this hornwort species on molecular and biochemical levels (Petersen 2003; Pezeshki 2016; Wohl and Petersen 2020a, b) showing the conservation of the general phenylpropanoid pathway also in non-seed plants as already proposed previously (Labeeuw et al. 2015; de Vries et al. 2017; Renault et al. 2019). Furthermore, in all the above-mentioned compounds (except anthocerodiazonin), a hydroxyphenyllactic acid moiety is incorporated which is derived from l-tyrosine by tyrosine aminotransferase (TAT) and hydroxyphenylpyruvic acid reductase (HPPR), two enzymes also present in *Anthoceros agrestis* and currently under investigation in our laboratory (Busch and Petersen (2021) and own unpublished results). The further biosynthesis of RA (Fig. 1) (and putatively also of anthocerotonic and megacerotonic acids) requires a hydroxycinnamoyltransferase to link the two precursors, 4-coumaroyl-CoA and 4-hydroxyphenyllactic acid, and a hydroxylase to finalize the substitution patterns at the aromatic rings as described for *Coleus blumei* (Lamiaceae) by Berger et al. (2006) and Eberle et al. (2009) or for *Phacelia campanularia* (Boraginaceae) by Levsh et al. (2019).

The first appearance of true phenolic compounds in the plant kingdom has been related to the formation of cuticle-like structures and thus to the protection of the land-colonizing plants against desiccation. Therefore, the phenylpropanoid pathway has been regarded as essential for plant terrestrialization, not only to protect against drought but also to provide a screen against UV radiation and to protect against pathogens and herbivores as well as to enable vertical growth by providing lignin for cell wall reinforcement (Weng and Chapple 2010; de Vries et al. 2021). In *Physco-mitrium patens*, the formation of caffeoylthreonic acid from *p*-coumaroylthreonic acid by CYP98A34 (the only member of the CYP98 group in this plant species) has been reported to be essential for the formation of a phenolic-containing cuticle while shikimic or quinic esters were not detected in first investigations (Renault et al. 2017). However, the CYP98A34 hydroxylating *p*-coumaroylthreonic acid was later found to prefer *p*-coumaroylanthranilic acid as substrate, while *p*-coumaroylshikimic acid was less accepted and *p*-coumaroyl-2-threonic and *p*-coumaroyl-4-threonic acids were only poorly converted. Similar results were shown for CYP98A38 from the lycopod *Selaginalla moel-lendorffii* and CYP98A104 from the fern *Pteris vittata* (Alber et al. 2019). These observations made a pathway to caffeic acid derivatives leading through phenolamides probable, while in seed plants, generally *p*-coumaroylshikimic acid was shown to be the responsible intermediate in the monolignol biosynthetic pathway (Schoch et al. 2001). The missing ability of *Physcomitrium* CYP98A34 to complement a CYP98A3 knockout mutant of *Arabidopsis thaliana* furthermore made an in vivo formation of caffeoylshikimic acid by the moss cytochrome P450 improbable (Alber et al. 2019). Findings by Kriegshauser et al. (2021), however, showed that the main intermediates formed by *Physcomitrium patens* hydroxycinnamoyltransferase *Pp3c2_29140* are hydroxycinnamoyl shikimic acid esters and not amides and thus a pathway similar to the one in seed plants was suggested (see Fig. 1). In seed plants, the main pathway towards higher substituted hydroxycinnamic acid derivatives (e.g., caffeic, ferulic or sinapic acids) and monolignols leads via a *p*-coumaric acid ester with shikimic acid (Hoffmann et al. 2003) which is then hydroxylated by a member of the CYP98 family to caffeoylshikimic acid (Schoch et al. 2001) after which caffeic acid (Vanholme et al. 2013; Saleme et al. 2017) or caffeoyl-CoA is released (Hoffmann et al. 2003) for further biosyntheses. Silencing of caffeoylshikimic acid esterase has been shown to alter lignification in poplar and to lead to an accumulation of caffeoylshikimic acid (Vanholme et al. 2013; Saleme et al. 2017). Reinvestigation of the substrate specificities of *Physcomitrium patens* hydroxy- cinnamoyltransferase (encoded by *Pp3c2_29140*) and CYP98A34 finally led to the conclusion that also mosses synthesize their caffeic acid metabolites via caffeoylshikimic acid (Kriegshauser et al. 2021).

Hydroxycinnamoyltransferases of the BAHD superfamily are known for their sometimes astonishing substrate promiscuity (Landmann et al. 2011; Sander and Petersen 2011) and some structural aspects important for substrate selection have been investigated (Chiang et al. 2018). It is assumed, that gene duplication and neo-functionalization events lead to the extended numbers of hydroxycinnamoyltransferases in angiosperms (Tuominen et al. 2011) with varying degrees of substrate specificity. While many hydroxycinnamoyl-CoA:shikimic/quinic acid hydroxycinnamoyltransferases accept shikimic as well as quinic acid (Schoch et al. 2001; Mahesh et al. 2007; Moglia et al. 2009), the hydroxycinnamoyl-CoA:shikimic acid hydroxycinnamoyltransferase (CbHST) from *Coleus blumei* accepts shikimic acid but not quinic and hydroxyphenyllactic acids, while hydroxycinnamoyl-CoA:hydroxyphenyllactic acid hydroxycinnamoyltransferase (better known as RA synthase (RAS); CbRAS)—involved in RA biosynthesis—from the same species accepts hydroxyphenyllactic acid derivatives and does not catalyze esterification of shikimic or quinic acids. However, also other acceptor substrates were accepted, e.g., 3-hydroxyanthranilic acid (but not anthranilic acid), 3-hydroxybenzoic, 2,3-dihydroxybenzoic and 3-aminobenzoic acids by CbHST and d-DOPA, d-tyrosine and d-phenylalanine by CbRAS (Sander and Petersen 2011). Comparable substrate promiscuities have been reported for RAS from *Lavandula angustifolia* by Landmann et al. (2011) and for three partially specific hydroxycinnamoyltransferases from *Phacelia campanularia* (Levsh et al. 2019) as well as the hydroxycinnamoyltransferase encoded by *Pp3c2_29140* from *Physcomitrium patens* (Kriegshauser et al. 2021). Notably, these hydroxycinnamoyltransferases catalyze ester as well as amide formation.

The finalization of the substitution pattern of *p*-coumaric acid esters and amides is catalyzed by cytochrome P450 monooxygenases of the CYP98 family. As reported by Alber et al. (2019), only one copy of CYP98 is present in bryophytes, lycophytes, monilophytes and gymnosperms, but multiple CYP98 family members evolved in angiosperms with varying substrate acceptance spectra. The single copy of CYP98 in the above-mentioned taxa may not in all cases be related to the biosynthesis of lignin since lignin is missing in bryophytes, and the lycophyte *Selaginella* uses a different cytochrome P450 (CYP788A1) for the formation of monolignols (Weng et al. 2010; Alber et al. 2019). It seems that CYP98 monooxygenases can accept a wide range of *p*-coumaric acid esters or amides, thus mainly showing specificity towards the hydroxycinnamoyl moiety, while, however, *p*-coumaric acid itself is generally no or only a very poor substrate (Alber et al. 2019). It must be noted, however, that a broad substrate range has only been tested for very few of these hydroxylases.

We here report on a hydroxycinnamoyltransferase from the hornwort *Anthoceros agrestis* forming esters between a hydroxycinnamic acid and shikimic acid or 2,3-dihydroxybenzoic acid as well as an amide between the hydroxycinnamic acid and 3-hydroxyanthranilic acid. The second enzyme reported here is the single CYP98 monooxygenase from the same organism introducing the 3-hydroxyl group into several *p*-coumaric acid esters and amides.

## Materials and methods

### Plant cell cultures

*Anthoceros agrestis* Paton cell suspension cultures originated from a callus culture from Binding and Mordhorst (1991) and were cultivated as described previously (Petersen 2003).

### Preparation of cDNA and amplification of partial sequences of Aa*HCT6* and Aa*CYP98*

Isolation of RNA was performed according to Chomczynski and Sacchi (1987). After checking RNA integrity, cDNA was prepared with the RevertAid First Strand cDNA Synthesis Kit (ThermoFisher). Internal PCR primers were designed based on *Anthoceros punctatus* scaffold 3426 (Aa*HCT6*) and on *Anthoceros agrestis* scaffold 26091 (Aa*CYP98*) after searching the *Anthoceros punctatus* and *Anthoceros agrestis* genome databases with the rosmarinic acid synthase (ENA: AM283092) and the CYP98A14 (ENA: AJ427452) sequences from *Coleus blumei*, respectively (Steven Kelly, University of Oxford, UK, personal communication; Peter Szövenyi, Universität Zürich, Switzerland, personal communication; Szövényi et al. 2015). All primers were synthesized by Eurofins Genomics and are listed in Suppl. Table S1.

For the amplification of partial sequences encoding AaCYP98 and AaHCT6, PCR assays of 25 µl were conducted with up to 0.2 µg cDNA, 0.5 µl 10 mM dNTP mix, 0.5 µl of each primer (Ap3426_f + Ap3426_r 100 µM each; Aa26091_f + Aa26091_r 10 µM each), 3.0 µl 25 mM MgCl_2_, 5.0 µl 5 × GoTaq buffer and 0.1 µl GoTaq polymerase (5 U/µl, Promega). The following programs were used for Aa*CYP98* (a) or Aa*HCT6* (b): 1 cycle 94 °C 120 s, 52 °C 60 s (a) / 60 °C 30 s (b), 70 °C 90 s; 38 cycles 94 °C 30 s, 52 °C 60 s (a) / 60 °C 30 s (b), 70 °C 90 s; 1 cycle 94 °C 120 s, 52 °C 60 s (a) / 60 °C 30 s (b), 70 °C 600 s. Amplification of the target sequences was checked by gel electrophoresis on a 0.7% agarose gel in TAE buffer (40 mM Tris, 20 mM acetic acid, 1 mM EDTA) using the GeneRuler DNA Ladder Mix (ThermoFisher) as marker. The PCR products were isolated with the NucleoSpin Gel and PCR Clean-up Kit (Macherey–Nagel). After ligation into pDrive (Qiagen), transformation and multiplication in *E. coli* EZ (Qiagen), the sequences were determined (Microsynth Seqlab).

### RACE-PCR and amplification of full-length Aa*HCT6* and Aa*CYP98* sequences

According to the sequences determined in the previous step, rapid amplification of cDNA ends (RACE)-PCR primers (Aa3426_5′R, Aa3426_3′R; Aa26091_5′R, Aa26091_3′R; Suppl. Table S1) was designed. RACE-PCR cDNA synthesis, PCR and InFusion^®^-cloning (only for Aa*CYP98*) were performed using the SMARTer^®^ RACE 5′/3′ kit (Takara/Clontech). After purification of the PCR products (NucleoSpin Gel and PCR Clean-up Kit, Macherey–Nagel), insertion into pRACE (Aa*CYP98*) or ligation into pDrive (for Aa*HCT6*), *E. coli* EZ were transformed and grown overnight. The plasmid was isolated and the sequence determined (Microsynth Seqlab). For amplification of the full-length sequence of Aa*HCT6*, primers with restriction sites for NdeI (Aa3426_VL-f) in the forward and BamHI (Aa3426_VL-r) in the reverse primer were designed for integration into the expression vector pET-15b (Novagen). For amplification and integration of the full-length sequence of Aa*CYP98* into the expression vector pESC-URA (Agilent Technologies), the sequence was equipped with restriction sites for EcoRI (5′) and NotI (3′) and the stop codon was removed. PCR reactions of 25 µl were performed as above, but using Pfu-Polymerase^®^ (2 U/µl; Promega; Aa*CYP98*) or Phusion polymerase (NEB) and the respective buffers using the same PCR program as above, but with annealing temperatures of 64 °C for Aa*HCT6* and 56 °C for Aa*CYP98*. After ligation of the purified PCR product into pDrive, *E. coli* EZ cells were transformed, grown in LB medium with 100 µg/ml ampicillin overnight at 37 °C and the sequence was determined (Microsynth Seqlab) after plasmid isolation. The Aa*HCT6* (= Aa3426) full-length sequence (GenBank MW248389) was released from pDrive by restriction digest with NdeI and BamHI and ligated into the vector pET-15b into the same restriction sites. The Aa*CYP98* (= Aa26091) full-length sequence (GenBank MT119883) was cut from the cloning plasmid with EcoRI and NotI and integrated into the respective restriction sites of the multiple cloning site I (MSCI) of the vector pESC-URA. By integration, Aa*HCT6* was attached to the sequence encoding an *N*-terminal 6xHis-tag already present on the expression vector pET-15b and Aa*CYP98* was attached to the sequence encoding a *C*-terminal FLAG-tag of the expression vector pESC-URA. The sequences were again checked for correctness (Microsynth Seqlab) after transformation (*E. coli* EZ), growth in LB medium with 100 µg/ml ampicillin at 37 °C overnight and plasmid isolation.

### Construction of phylogenetic trees

For phylogenetic analysis, the translated amino acid sequences of AaHCT6 or AaCYP98 were aligned with other amino acid sequences from different species using the Maximum Likelihood method of the MEGA7 software package. The robustness of the branch structure was evaluated by bootstrap analysis (1000 replicates). The sequences for the phylogenetic trees were accessed from the BRENDA enzyme database, NCBI searches and Alber et al. (2019) (see Suppl. Figs. S2 and S3 for accession numbers).

### Protein expression and isolation of AaHCT6

For expression of Aa*HCT6*, *E. coli* SoluBL21 (Amsbio) bacteria were transformed with the pET-15b vector carrying the full-length sequence of Aa*HCT6*. The bacteria were cultivated overnight in 2 ml LB-medium containing 100 µg/ml ampicillin at 37 °C, 220 rpm and then transferred into a 250 ml Erlenmeyer flask containing 100 ml TB-medium with 100 µg/ml ampicillin (for media composition see Lessard 2013). The bacteria were cultivated at 220 rpm and 37 °C to an optical density at 600 nm (OD_600_) of 0.4–0.6. Expression was then induced by addition of isopropyl-β-d-1-thiogalactopyranoside (final concentration 1 mM). The cultures were incubated for 24 h at 180 rpm and 25 °C. Bacteria were harvested by centrifugation for 15 min at 8000*g*, re-suspended in 4 ml per g fresh weight potassium phosphate buffer (KP_i_ 0.1 M, pH 8.0) and incubated on ice with 50 mg lysozyme for 30 min. The bacteria were then disrupted by ultra-sonication for 1 min at 0 °C and centrifuged for 15 min at 8000*g*. The supernatant was collected as crude protein extract. Purification of the AaHCT6 protein carrying an *N*-terminal 6xHis-tag was attempted by metal-chelate chromatography with Ni–NTA resin (Novagen) followed by desalting through PD-10 columns (GE Healthcare) but resulted in severe activity losses and was therefore not routinely applied.

### Codon optimization, protein expression and isolation of AaCYP98

At first, *Saccharomyces cerevisiae* BY4742 (ATCC 201389) was transformed with the native Aa*CYP98* coding sequence in pESC-URA, but after expression, AaCYP98 protein was not detectable by Western blot analysis. For this reason, we decided to codon-optimize Aa*CYP98* (= coAa*CYP98*) for the expression in yeast; this was carried out commercially by GeneCust (Boynes, France). The sequence was equipped with restriction sites for EcoRI (5′) and NotI (3′) and the stop codon was removed. The codon-optimized sequence was delivered in the vector pUC57 which was used to transform *E. coli* EZ for plasmid multiplication. The codon-optimized Aa*CYP98* sequence was released from the vector with EcoRI and NotI and integrated into the suitable restriction sites of the multiple cloning site I (MSCI) of the vector pESC-URA. Additionally, a sequence encoding a NADPH:cytochrome P450 reductase (CPR) from *Coleus blumei* (Cb*CPR*) characterized by Eberle et al. (2009) was ligated into multiple cloning site II (MCSII) into the restriction sites for BamHI and SalI, alternatively MSCII was left empty. *Saccharomyces cerevisiae* BY4742 was transformed with the plasmids containing only coAa*CYP98* or coAa*CYP98* + Cb*CPR* essentially as described by Gietz and Schiestl (2007) and cultivated on SCD_-ura_ plates (Dymond 2013). Plasmid uptake was checked by colony-PCR: single colonies were picked and suspended in 100 μl 200 mM lithium acetate, 1% SDS solution and incubated for 5 min at 70 °C. For DNA precipitation, 300 µl ethanol (96%) was added and mixed vigorously. The samples were centrifuged for 3 min at 15,000*g* and the resulting pellet was washed with 300 µl 70% ethanol. Afterwards, the tubes were centrifuged again for 3 min at 15,000*g* and the pellet was dissolved in 50 µl H_2_O. 2 µl of the solution was used in a PCR reaction as described above with GoTaq polymerase and specific primers for the respective multiple cloning sites (MCSI_f + MCSI_r or MCSII_f + MCSII_r; Suppl. Table S1). Amplification was checked by agarose gel electrophoresis. For protein expression, a small amount of transformed yeast cells was transferred from a SCD_-ura_ plate to 100 ml liquid SCD_-ura_ in a 500-ml baffled flask and incubated for 48 h at 30 °C at 160 rpm. Then the cells were transferred to 100 ml SCG_-ura_ medium supplemented with 200 µM 5-aminolevulinic acid and 12.5 mg l^−1^ FeSO_4_ after cell collection by centrifugation for 5 min at 3000*g* at 4 °C. The cultures were incubated for another 24 h at 30 °C and 160 rpm and the cells then collected by centrifugation for 5 min at 3000 g and 4 °C. The cell pellet was re-dissolved in 10 ml buffer (0.1 M Tris–HCl pH 7.0, 1 mM dithiothreitol (DTT), 1 mM sodium diethyldithiocarbaminate (DIECA)) and left on ice for 5 min. To remove the remains of the medium, the cells were again sedimented for 5 min at 3000*g* (4 °C) and the supernatant was discarded. The pellet was re-suspended in 1.5 ml buffer (as above) and transferred to a 7-ml tube with 2.5 g glass beads (~ 0.5 mm ø). Disintegration was done by vigorous shaking in a benchtop homogenizer (Minilys^®^) at 4000 rpm for 30 s, followed by cooling on ice for 30 s and 6–8 repetitions. The supernatant was removed after the glass beads had settled and glass beads were washed with 0.5 ml buffer. The crude protein extract was obtained after the supernatants were pooled and centrifuged for 20 min at 5000*g* (4 °C). The extract was used directly for the determination of enzyme activities. Samples for SDS-PAGE or Western blot analysis were stored at − 20 °C.

### SDS-PAGE and western blot

Protein extracts were subjected to SDS-PAGE essentially according to Laemmli (1970). After electrophoresis, the gel was either stained with Coomassie Brilliant Blue R250 or Western blotting was performed basically as specified by Mahmood and Yang (2012), but applying the Towbin et al. (1979) buffer system. The expressed proteins were detected with mouse anti-6xHis-tag (ThermoFisher, MA1-21315) or mouse anti-FLAG-tag (ThermoFisher, MA1-91878) monoclonal antibodies, followed by goat anti-mouse antibodies conjugated to alkaline phosphatase (Life Technologies, A16087) as secondary antibody. Staining was performed with nitro blue tetrazolium chloride (NBT)/5-bromo-4-chloro-3-indolylphosphate (BCIP) essentially following standard protocols (https://www.sysy.com/protocols/westernblot-ap-detection). Here the alkaline phosphatase coupled to the secondary antibody dephosphorylates BCIP followed by oxidation and indoxyl dimerization to dibromodichloroindigo. Additionally, reduction equivalents reduce NBT to its formazan. Both insoluble pigments can be detected by their purple coloration (Sambrook and Russell 2001).

### Production of substrates for AaHCT6 and AaCYP98

#### Chemical synthesis of *p*-coumaroyl-CoA and caffeoyl-CoA

The synthesis of hydroxycinnamoyl-CoA thioesters followed essentially the protocol of Stöckigt and Zenk (1975). The CoA-esters were purified using Chromabond C18ec cartridges (Macherey–Nagel) with 4% aqueous ammonium acetate to rinse the column and water for elution (Beuerle and Pichersky 2002). The CoA-ester concentrations were determined photometrically using the absorption maxima and molecular extinction coefficients published by Stöckigt and Zenk (1975) and Zenk (1979).

#### Enzymatic synthesis of *p*-coumaroylshikimic, *p*-coumaroylquinic and *p*-coumaroyl-3-hydroxyanthranilic acids

*p*-Coumaroylshikimic, *p*-coumaroylquinic and *p*-coumaroyl-3-hydroxyanthranilic acids were produced enzymatically using heterologously synthesized hydroxycinnamoyltransferases from *Anthoceros agrestis* and *Sarcandra glabra* (from our laboratory collection) with *p*-coumaroyl-CoA and the corresponding acid as substrates*.* Assays were composed as follows: 100 µl buffer (0.1 M potassium phosphate buffer (KP_i_) pH 7.0), 10 µl *p*-coumaroyl-CoA (2.5 mM), 5 µl ascorbic acid (12.5 mM), 5 µl substrate (10 mM) and 5 µl protein preparation. All enzyme assays were incubated up to 3 h at 30 °C and then heated to 95 °C for 5 min to inactivate the enzymes. Products were used directly for qualitative assays without further purification. Alternatively, *p*-coumaroylshikimic acid was purified by HPLC using a semi-preparative Equisil ODS column (250 × 8 mm; pre-column: 20 × 8 mm; particle size 5 µm) and isocratic elution with 35% aqueous methanol containing 0.3% acetic acid at a flow rate of 4 ml/min and detection at 312 nm. Samples were stored at -20 °C until use.

#### Isolation of *p*-coumaroyl-4′-hydroxyphenyllactic acid and caffeoyl-4′-hydroxyphenyllactic acid from suspension cultures of *Melissa officinalis*

The precursors of RA, *p*-coumaroyl-4′-hydroxyphenyllactic acid and caffeoyl-4′-hydroxyphenyllactic acid, were isolated from *Melissa officinali*s suspension cultures treated with the cytochrome P450 inhibitor tetcyclacis (BASF). 100 μM tetcyclacis (in acetone, filter-sterilized) was added to a four-day-old suspension culture of *Melissa officinalis* (cultivated in CB2 medium (Petersen and Alfermann 1988) at 25 °C in the dark at 100 rpm) and the cells were incubated for another 7 days under the same conditions. The cells were harvested by vacuum filtration and stored at − 20 °C until further use. 0.01% aqueous H_3_PO_4_ was heated to 100 °C, added to the cells (1 ml/g cell fresh weight) and the mixture was incubated at 80 °C for 20 min. The cells were filtered off and the filtrate was extracted three times with 25 ml ethyl acetate (EtOAc) each. The organic phases were collected and evaporated and the residue was re-suspended in 2 ml EtOAc. The cell extract was applied to a preparative silica gel TLC plate (silica gel 60 F_254_) and developed in EtOAc:chloroform:formic acid (5:4:1, by vol.) as mobile phase. The expected bands were scraped off and eluted four times with 1 ml methanol each. The concentration was determined photometrically using the following absorption maxima and molecular extinction coefficients (*p*-coumaroyl-4′-hydroxyphenyllactic acid 312 nm, ε = 11.94 mM^−1^ cm^−1^; caffeoyl-4′-hydroxyphenyllactic acid 328 nm, ε = 9.15 mM^−1^ cm^−1^).

#### Chemical synthesis of *p*-coumaroylanthranilic acid, *p*-coumaroyl-3-hydroxyanthranilic acid and *p*-coumaroyltyramine

*p*-Coumaroylanthranilic acid and *p*-coumaroyl-3-hydroxyanthranilic acid were synthesized essentially according to Alber et al. (2019). For the synthesis of *p*-coumaroyl-tyramine, one equivalent (0.3 g) *p*-coumaric acid was dissolved in 10 ml acetonitrile, then triethylamine (0.642 ml, 2.5 equivalents) and 1-ethyl-3-(3-dimethylaminopropyl)carbodiimide × HCl (0.420 g, 1.2 equivalents) were added and the solution was stirred at 0 °C for 30 min. After addition of 1 equivalent (0.250 g) tyramine, the mixture was stirred at room temperature overnight. The solvent was evaporated and the remaining solid was re-suspended in 50 ml water and extracted three times with 50 ml EtOAc. After evaporation of the organic solvent, the residue was purified by column chromatography using silica gel as solid phase with isocratic elution using EtOAc:cyclohexane (3:1, v/v).

All products were analyzed by ^1^H-NMR, *p*-coumaroyl-3-hydroxyanthranilic acid was additionally analyzed by ^13^C-NMR (Suppl. Table S2). Data unequivocally show that the product of the chemical synthesis is an amide of *p*-coumaric acid and 3-hydroxyanthranilic acid and not an ester.

*p*-Coumaroyl-2-threonic acid was kindly provided by Dr. Hugues Renault, Université de Strasbourg.

### Standard assay for AaHCT6 and reaction kinetics

AaHCT6 standard enzyme assays with shikimic acid were performed in 1.5 ml reaction tubes containing 105 µl potassium phosphate buffer (KP_i_) buffer pH 7.0, 5 µl 12.5 mM ascorbic acid, 5 µl 2.5 mM *p*-coumaroyl- or caffeoyl-CoA, 5 µl 10 mM shikimic acid and 5 µl crude protein extract (appr. 5 µg protein), assays with 3-hydroxyanthranilic acid were composed similarly but contained 105 µl 1 M KP_i_ buffer pH 9.0, 5 µl 12.5 mM ascorbic acid, 5 µl 2.5 mM *p*-coumaroyl- or caffeoyl-CoA, 5 µl 10 mM 3-hydroxyanthranilic acid (in 1 M HCl) and 5 µl crude protein extract (appr. 0.5 µg protein). The reactions were pre-warmed at 30 °C for 15 min and then started by addition of the protein. Incubation lasted for 5 min and the reaction was stopped by addition of 20 µl 6 M HCl for assays with shikimic acid and 60 µl 6 M HCl for assays with 3-hydroxyanthranilic acid. The reaction products were extracted twice with 500 µl EtOAc each. After evaporation of the solvent, the residues were re-dissolved in 100 µl of the respective HPLC mobile phase (see below). The reaction products were analyzed and quantified by HPLC using an Equisil ODS column (250 × 4 mm; pre-column: 20 × 4 mm; particle size 5 µm) and isocratic elution with 35% (products with shikimic acid) or 50% (products with 3-hydroxyanthranilic or 2,3-dihydroxybenzoic acids) aqueous methanol containing 0.01% H_3_PO_4_ at a flow rate of 1 ml/min and detection at 312 nm for *p*-coumaric acid derivatives and 333 nm for caffeic acid derivatives. For the determination of kinetic values, the substrates and substrate concentrations were varied (for details Suppl. Table S3). Data were analyzed with the GraphPad Prism 5 software using the Michaelis–Menten, Lineweaver–Burk (not shown) and Hanes–Woolf models.

### Standard assay for AaCYP98 and reaction kinetics

Standard assays for AaCYP98 in 1.5 ml reaction vials contained 100 µl crude protein extract (0.2 mg protein), 7.5 µl 10 mM substrate (in 50% methanol) or HCT-enzyme assays (see above), 12.5 µl 50 mM NADPH and 5 µl buffer (0.1 M Tris–HCl pH 7.0, 1 mM DTT, 1 mM DIECA). Assays were mixed vigorously and incubated for 120 min at 30 °C under shaking at 1200 rpm in an Eppendorf Thermomixer. The incubation time for kinetic assays was kept at 5 min (*p*-coumaroyl-3-hydroxyanthranilic acid) and 10 min (*p*-coumaroylanthranilic acid and *p*-coumaroyltyramine) or 30 min (*p*-coumaroylshikimic acid) after having ensured the linearity of product formation at the lowest used substrate concentration at this time point. The reaction was stopped by addition of 50 µl 6 M HCl. The assays were extracted twice with 500 µl EtOAc each and the EtOAc extracts combined and evaporated. The residues were re-dissolved in 50 µl methanol containing 0.01% H_3_PO_4_ (85%) and centrifuged after addition of 50 µl aqueous 0.01% H_3_PO_4_. Quantification and analysis of the reaction product were performed by HPLC (injection volume 50 µl) or LC–MS (injection volume 10 µl). Analysis by HPLC was performed using a Hypersil ODS column (250 × 4 mm; pre-column: 20 × 4 mm; particle size 5 µm) and isocratic elution with 45% or 50% aqueous methanol containing 0.01% H_3_PO_4_ at a flow rate of 1 ml/min and detection at 333 nm. LC was performed on an Agilent Technologies HPLC 1260 with a Multospher 120 RP18 column (250 × 2 mm; particle size 5 μm) using a solvent system of A = 0.1% (v/v) aqueous formic acid, B = acetonitrile with 0.1% (v/v) formic acid at a flow rate of 0.25 ml/min and a temperature of 20 °C using the following gradient: 0–40 min 5% B → 100% B; 40–45 min 100% B; 45–45.10 min 100% B → 5% B; 45.10–55 min 5% B. Detection was performed with the mass spectrometer micrOTOF-Q III with ESI source (Bruker Daltonics) calibrated with 5 mM CHNaO_2_ using the negative mode. Data from kinetic analysis are deduced from at least three independent protein isolations with three technical replicates for each substrate concentration and were analyzed with the GraphPad Prism 5 software using the Michaelis–Menten, Lineweaver–Burk (not shown) and Hanes–Woolf models.

## Results

### Isolation and analysis of a cDNA encoding a hydroxycinnamoyltransferase (AaHCT6) from *Anthoceros agrestis*

Searching the genome database of *Anthoceros punctatus* (Kelly, personal communication), a close relative of *Anthoceros agrestis*, with the protein sequence of *Plectranthus scutellarioides* RAS (UniProtKB: A0PDV5) resulted in the identification of several putative BAHD hydroxycinnamoyltransferase sequences. The sequence with the highest identity with the query sequence, scaffold 3426, was further used for this study. PCR with cDNA from *Anthoceros agrestis* suspension cultures and primers directed against this partial putative BAHD acyltransferase scaffold resulted in a partial sequence (1016 bp) similar to known hydroxycinnamoyltransferase sequences. RACE-PCR completed the full-length sequence comprising 1305 bp encoding a protein of 434 amino acid residues, which we named AaHCT6. The Aa*HCT6* sequence was deposited in GenBank under the accession number MW248389. The translated amino acid sequence displayed identities of 55–59% and similarities of 78–80% to hydroxycinnamoyltransferases from di- and monocotyledonous plants (FASTA). The similarity/identity (EMBOSS Needle) to RA synthases from *Melissa officinalis* (UniProtKB: G0LD36) and *Plectranthus scutellarioides* (UniProtKB: A0PDV5) were at 62.6/46.2% and 62.6/44.8%, respectively. Due to the long loop found in *Physcomitrium patens* HCT *Pp3c2_29140* (Kriegshauser et al. 2021), the identity to this sequence is only at 47.4%. The translated protein had a molecular mass of 47.5 kDa and an isoelectric point of 6.22. Conserved sequence motifs for BAHD acyltransferases, such as HxxxDG and DFGWG (St. Pierre and De Luca 2000), are present as well as the “arginine handle” described by Chiang et al. (2018) to be essential for substrate recognition (see boxes in Suppl. Fig. S4). This motif is altered in RAS from *Melissa officinalis* (Weitzel and Petersen 2011) where the Arg residue is replaced by Gln. The conserved amino acid residues Thr and Trp located between the “arginine handle” and the DFGWG motif were reported to be involved in substrate binding and catalysis besides the catalytic His residue of the HxxxDG motif (Chiang et al. 2018). The conservation coloring in Suppl. Fig. S4 shows the rather high conservation within the two protein domains, but distinct differences in the lengths of the loops connecting these two domains as also described by Kriegshauser et al. (2021). This loop is much less extended in AaHCT6 than in hydroxycinnamoyltransferases from *Physcomitrium patens* or *Marchantia paleacea* and thus resembles more seed plant hydroxycinnamoyltransferases.

### Isolation and analysis of a cDNA encoding CYP98 from *Anthoceros agrestis*

Based on PCR primers directed against a partial putative *CYP98* sequence from *Anthoceros agrestis* (scaffold 26091; P. Szövenyi, personal communication), an internal 1050 bp fragment was isolated and sequenced. This fragment showed high similarities to other plant *CYP98* sequences. RACE-PCR was used to amplify the cDNA ends. The full open reading frame consisted of 1515 bp. This sequence can be translated to a 56.7 kDa protein with 505 amino acid residues and an isoelectric point at pH 9.19. The *CYP98* sequence from *Anthoceros agrestis* was deposited in GenBank under the accession number MT119883 and was classified as CYP98A147 by Dr. David Nelson (cytochrome P450 homepage: https://drnelson.uthsc.edu/). As depicted in Suppl. Fig. S5, the amino acid sequence of AaCYP98 showed elements generally found in the canonic P450 monooxygenases, such as the proline-rich region (Bak et al. 2011), the PERF motif, the heme-binding cysteine motif and the threonine-containing binding pocket motif (Schuler 1996; Mizutani et al. 1997; Chapple 1998). On amino acid level, AaCYP98 was analyzed by BLASTp and showed highest identity (71%) to CYP98 from *Physcomitrium patens.* The highest identity (65%; FASTA) with a seed plant CYP98 was to CYP98A3 from *Arabidopsis thaliana* (UniProtKB: O22203) which catalyzes the *meta*-hydroxylation of *p*-coumaroylshikimic or quinic acid esters (Schoch et al. 2001). The similarity/identity (EMBOSS Needle) on amino acid level to CYP98A14 from *Plectranthus scutellarioides* involved in RA biosynthesis was at 75.4/63.1%.

### Phylogenetic analyses of AaHCT6 and AaCYP98 amino acid sequences

Phylogenetic trees were constructed with the MEGA7 software package for both, AaHCT6 and AaCYP98 amino acid sequences using the maximum likelihood algorithm (Suppl. Figs. S2 and S3).

In the phylogenetic analysis (Suppl. Fig. S2) based on amino acid sequences of BAHD hydroxycinnamoyltransferases, AaHCT6 turns up in the same sub-branch as hydroxy-cinnamoyltransferases from the moss *Physcomitrium patens* and two liverworts as well as two hydroxycinnamoyltransferases from the lycophyte *Selaginella moellendorffii*. The enzymes from this branch that were already tested for substrate acceptance convert shikimic, quinic and (hydroxy-)anthranilic acids. The phylogenetic tree shows two major branches. One branch contains two groups of dicotyledonous plant hydroxycinnamoyltransferases accepting shikimic and/or quinic acids. These two groups are separated by hydroxy-cinnamoyltransferases from grasses, gymnosperms, lycophytes and bryophytes. The phylogenetic analysis further shows that the relationships are not ordered according to the evolutionary level. The second major branch contains hydroxycinnamoyltransferases from seed plants as well as bryophytes with a very diverse substrate spectrum, among them those involved in RA biosynthesis. Anthranilic, malic and piscidic acids as well as spermine/spermidine, tetrahydroxyhexanedioic acid and fatty acids also occur as acceptor substrates for hydroxycinnamoyltransferases in this branch besides shikimic and quinic acids which are only rarely represented here. The observed scheme underlines the general hypothesis that substrate diversification has occurred several times during the evolution of hydroxycinnamoyltransferases and that hydroxycinnamoyltransferases with the same substrate range have evolved independently.

AaCYP98 groups closest with the single CYP98 sequences from mosses (*Physcomitrium patens*, *Sphagnum fallax*) and the liverwort *Marchantia polymorpha* followed by the lycophyte *Selaginella moellendorffii* and the fern group represented by *Pteris vittata* and *Blechnum spicant* (Suppl. Fig. S3). These non-seed plant CYP98 members group on one branch which is neighbored by the branch of gymnosperm CYP98 members. The branch with monocotyledonous plants is divided into two sub-branches with the Poaceae CYP98s on one side-branch and *Phoenix dactylifera* (Arecaceae) on the other. CYP98s from dicotyledonous plants are found on a separate highly ramified main branch divided from all before-mentioned CYP98s. This can be explained by the already mentioned diversification of CYP98 in the angiosperms (Alber et al. 2019). Regarding substrate specificities, CYP98 enzymes metabolizing, e.g., shikimic acid or hydroxyphenyllactic acid esters appear on several separated branches indicating that substrate specificities have evolved several times independently as already postulated by Alber et al. (2019).

### Expression of *Anthoceros agrestis* HCT6 in *Escherichia coli* and characterization of hydroxycinnamoyltransferase activity

**Fig. 2:**
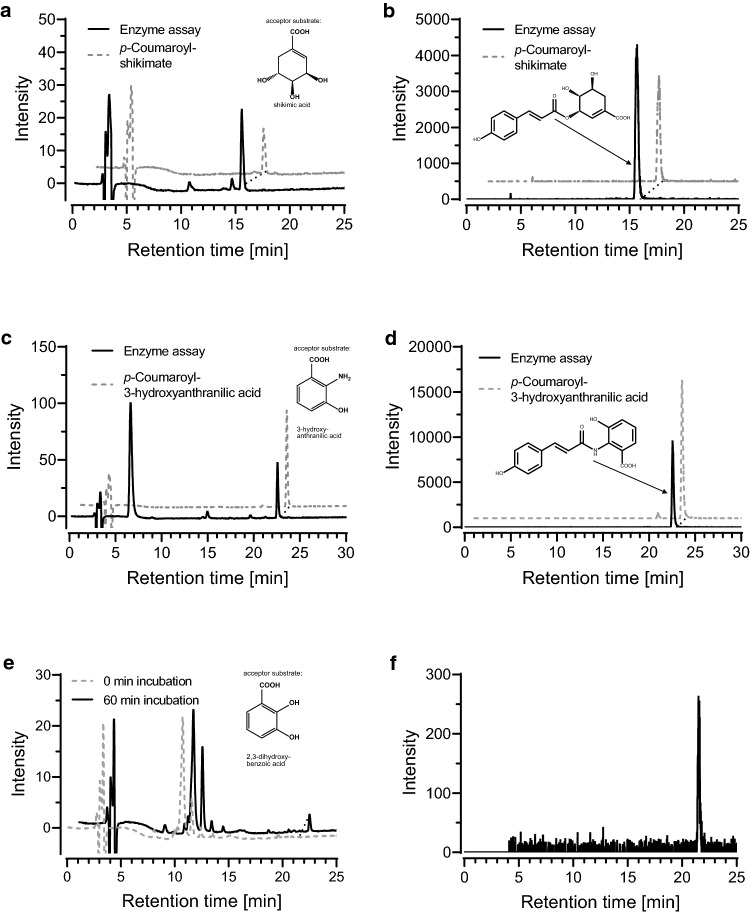
LC–MS analysis of *p*-coumaroylshikimic acid (**a** UV-chromatogram at 312 nm. **b** Extracted ion chromatogram (EIC) at *m/z* 319 [M-H]), *p*-coumaroyl-3-hydroxyanthranilic acid (**c** UV-chromatogram at 296 nm. **d** Extracted ion chromatogram (EIC) at *m/z* 298 [M-H]) and caffeoyl-2,3-dihydroxybenzoic acid (**e** UV-chromatogram at 296 nm. **f** Extracted ion chromatogram (EIC) at *m/z* 315 [M-H]) formed in enzyme assays by AaHCT6 (black line) and the respective standards (gray dashed line) or assay with reaction time 0 min for 2,3-dihydroxybenzoic acid (gray dashed line)

The full-length coding sequence of AaHCT6 with an *N*-terminally attached His-tag was ligated into the expression vector pET-15b and introduced into *E. coli* SoluBL21 for expression. The crude protein extract contained enzymatically active hydroxycinnamoyltransferases which did not appear in protein extracts from *E. coli* carrying the empty pET-15b vector. Thus, hydroxycinnamoyltransferase activity was assigned to the transgene. Unfortunately, attempts to purify the His-tagged protein by metal-chelate chromato-graphy resulted in almost complete loss of activity (an observation that was made for several different BAHD acyltransferases) and therefore the characterization experiments were performed with crude protein extracts. However, Western blot analyses clearly showed the presence of a His-tagged protein with the expected size missing in empty vector controls (Suppl. Fig. S6). First attempts aimed at identifying acceptor substrates using caffeoyl-CoA as hydroxycinnamoyl donor. AaHCT6 readily accepted shikimic acid together with *p*-coumaroyl-CoA and caffeoyl-CoA forming the respective esters. The identity of the formed *p*-coumaroylshikimic acid was verified by LC–MS analysis (Fig. 2a, b). Crude protein extracts from empty vector controls did not catalyze product formation (Suppl. Fig. S7). 3-Hydroxyanthranilic acid (but not anthranilic acid) was accepted as well; here, ester as well as amide formation would be possible. To verify the identity of the formed product, LC–MS analyses were performed which firstly indicated the correct mass at *m/z* 298 [M–H] and secondly displayed the identical retention time as the chemically synthesized *p*-coumaroyl-3-hydroxyanthranilic acid which was proven to be the amide (Fig. 2c, d). Ester formation was moreover observed with *p*-coumaroyl-CoA or caffeoyl-CoA and 2,3-dihydroxybenzoic acid (Fig. 2e, f). In total, we have tested 55 putative acceptor substrates (see Suppl. Table S4), among them 4-hydroxyphenyllactic acid and quinic acid, but only the above-mentioned three compounds resulted in product formation. The temperature and pH optima were determined with caffeoyl-CoA and shikimic acid to be at 35 °C and pH 7.3, respectively (Suppl. Fig. S8).

Kinetic data were determined for shikimic, 3-hydroxyanthranilic and 2,3-dihydroxybenzoic acids together with *p*-coumaroyl- and caffeoyl-CoA as hydroxycinnamoyl donors (Table 1 and Suppl. Fig. S9 and S10). The highest affinities were determined for *p*-coumaroyl-CoA as donor substrate (*K*_m_ 14.4 ± 1.4 µM with shikimic acid, *K*_m_ 29.3 ± 6.6 µM with 3-hydroxyanthranilic acid), caffeoyl-CoA displayed a slightly higher *K*_m_ value together with shikimic acid (19.2 ± 16.2 µM), whereas the *K*_m_ value with 3-hydroxyanthranilic acid was considerably higher (314.4 ± 28.7 µM). Interestingly, caffeoyl-CoA had a higher affinity with 2,3-dihydroxybenzoic acid as acceptor substrate (*K*_m_ caffeoyl-CoA 171.9 ± 13.2 µM, *p*-coumaroyl-CoA 388.7 ± 44.1 µM). Shikimic acid was accepted with a remarkably higher affinity than 3-hydroxyanthranilic acid and 2,3-dihydroxybenzoic acid, especially together with *p*-coumaroyl-CoA as donor substrate (*K*_m_ 24.6 ± 10.4 µM versus 153.1 ± 33.8 µM and 1025.9 ± 59.7 µM, respectively). With caffeoyl-CoA as donor substrate, the *K*_m_ values were quite high but in the same range (*K*_m_ 587.0 ± 26.5 µM for shikimic acid, 761.0 ± 74.7 µM for 3-hydroxyanthranilic acid, 686.7 ± 38.3 µM for 2,3-dihydroxybenzoic acid) indicating unfavorable substrate combinations. Since crude protein extracts had to be used for enzyme assays, real turnover numbers could not be calculated.

**Table 1:** Kinetic values of AaHCT6 for various acceptor and donor substrates in combination

*K*_m_ value for	With	MM: *K*_m_ [µM]	HW: *K*_m_ [µM]
*p*-Coumaroyl-CoA	Shikimic acid	14.4 ± 1.4	5.1 ± 1.3
Caffeoyl-CoA	Shikimic acid	19.2 ± 16.2	19.2 ± 17.1
*p*-Coumaroyl-CoA	3-Hydroxyanthranilic acid	29.3 ± 6.6	34.5 ± 5.2
Caffeoyl-CoA	3-Hydroxyanthranilic acid	314.4 ± 28.7	343.3 ± 15.6
*p*-Coumaroyl-CoA	2,3-Dihydroxybenzoic acid	388.7 ± 44.1	323.0 ± 31.5
Caffeoyl-CoA	2,3-Dihydroxybenzoic acid	171.9 ± 13.2	162.4 ± 15.7
Shikimic acid	*p*-Coumaroyl-CoA	24.6 ± 10.4	57.6 ± 13.0
Shikimic acid	Caffeoyl-CoA	587.0 ± 26.5	592.9 ± 35.5
3-Hydroxyanthranilic acid	*p*-Coumaroyl-CoA	153.1 ± 33.8	202.7 ± 34.2
3-Hydroxyanthranilic acid	Caffeoyl-CoA	761.0 ± 74.7	780.6 ± 67.7
2,3-Dihydroxybenzoic acid	*p*-Coumaroyl-CoA	1025.9 ± 59.7	1033.7 ± 57.4
2,3-Dihydroxybenzoic acid	Caffeoyl-CoA	686.7 ± 38.2	747.5 ± 50.8

Values were calculated with the help of Michaelis–Menten (MM) and Hanes–Woolf plots (HW). Mean values of at least six determinations ± standard error

### Expression of codon-optimized *Anthoceros agrestis CYP98* in *Saccharomyces cerevisiae* and characterization of CYP98 activity

Since protein formation was not detectable after expression of the native Aa*CYP98* sequence in yeast (data not shown), Aa*CYP98* was codon-optimized (coAa*CYP98*). After transformation of *Saccharomyces cerevisiae* BY4742 with pESC-URA harboring either only coAa*CYP98* (coAa*CYP98*) or coAa*CYP98* together with the coding sequence of the *Coleus blumei CPR* (coAa*CYP98* + Cb*CPR*), plasmid uptake was verified by colony-PCR (Suppl. Fig. S11a). With an expected size of approximately 1800 bp, coAa*CYP98* was detectable in all transformants. For transformants carrying also the *CPR* from *Coleus blumei*, colony-PCR was additionally performed using primers directed against the multiple cloning site II (MCSII) of the expression vector and a signal was detected at the expected size of around 2600 bp (Suppl. Fig. S11b). After expression, protein formation of coAaCYP98 was verified by Western blot analysis with anti-FLAG antibody, showing a band of the expected size (~ 60 kDa) (Suppl. Fig. S11a, b). Western blot analysis using anti-Myc antibody against CbCPR with a C-terminally Myc-tag was not attempted.

**Fig. 3:**
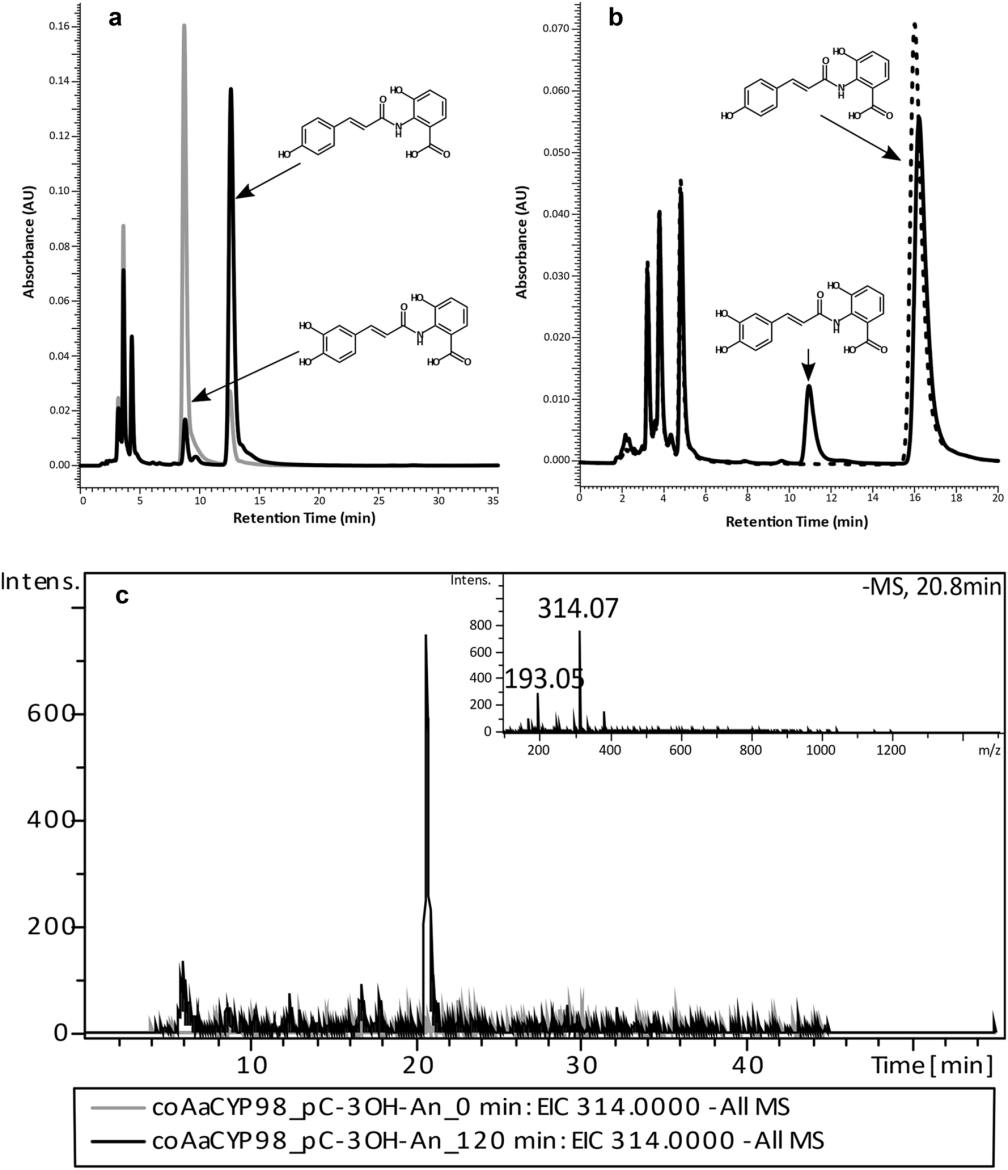
Activity of crude protein extract (200 µg protein) of coAaCYP98 or coAaCYP98 + CbCPR with *p*-coumaroyl-3-hydroxyanthranilic acid (pC-3-OH-An) analyzed by HPLC (333 nm) and LC–MS.** a** Enzyme assay with *p*-coumaroyl-3-hydroxyanthranilic acid after 120 min at 25 °C with either coAaCYP98 (black) or coAaCYP98 + CbCPR (gray). HPLC with isocratic elution with 50% methanol + 0.01% H_3_PO_4_. **b** *p*-Coumaroyl-3-hydroxyanthranilic acid incubated for 120 min at 25 °C with coAaCYP98 (solid line) or crude protein extract from the empty vector control (dashed line). HPLC with isocratic elution with 45% methanol + 0.01% H_3_PO_4_. **c** LC–MS analysis of coAaCYP98 incubated with *p*-coumaroyl-3-hydroxyanthranilic acid for 0 min (gray) or 120 min (black). HPLC with acetonitrile/water with 0.1% formic acid gradient elution. Chromatograms show the extracted ion chromatogram (EIC) of caffeoyl-3-hydroxyanthranilic acid in the negative mode. The exact mass of caffeoyl-3-hydroxyanthranilic acid is *m/z* 314.07 [M-H]

First activity assays were performed using crude protein extract from transformed yeast cells and *p*-coumaroyl-3-hydroxyanthranilic acid together with NADPH as substrates, since microsome preparation resulted in strongly reduced activities. HPLC analysis showed the formation of caffeoyl-3-hydroxyanthranilic acid for both coAaCYP98 and coAaCYP98 + CbCPR. However, co-expression of CPR resulted in 13-fold caffeoyl-3-hydroxyanthranilic acid formation (Fig. 3a, b). LC–MS analysis resulted in a peak at *m/z* 314.07 [M-H] corresponding to the exact mass of caffeoyl-3-hydroxyanthranilic acid (Fig. 3c). Protein extracts from BY4742 transformed with the empty vector were used as a negative control and displayed no product formation. *p*-Coumaroylanthranilic acid, *p*-coumaroyltyramine, *p*-coumaroylshikimic acid and *p*-coumaroyl-4′-hydroxyphenyllactic acid were converted as well by crude protein extracts of coAaCYP98 + CbCPR (Suppl. Fig. S12), but not by the less active coAaCYP98. Due to unknown reasons, both, *p*-coumaroylanthranilic acid and caffeoyl-3-hydroxyanthranilic acid split into two peaks in HPLC and LC–MS analysis (Suppl. Fig. S12). *p*-Coumaroylquinic acid, *p*-coumaroyl-2-threonic acid and caffeoyl-4’-hydroxyphenyllactic acid were not converted by AaCYP98.

*p*-Coumaroylanthranilic acid was used as substrate for the determination of temperature and pH optima. Suppl. Fig. S13 shows the pH and temperature optima for coAaCYP98 + CPR. They point out a temperature optimum at 30–35 °C and a pH-optimum around pH 7.

Kinetic data for AaCYP98 (coAaCYP98 + CPR) were determined for *p*-coumaroylanthranilic acid, *p*-coumaroyltyramine, *p*-coumaroyl-3-hydroxyanthranilic acid and for the co-substrate NADPH (Table 2; Suppl. Fig. S14). For determination of the kinetic values of *p*-coumaroylanthranilic acid, the areas for both product peaks were summed up. *p*-Coumaroylanthranilic and *p*-coumaroyl-3-hydroxyanthranilic acids were the substrates accepted with the highest affinities (*K*_m_ 6.4 ± 0.8 µM and 9.3 ± 0.7 µM, respectively), followed by *p*-coumaroyl-tyramine (*K*_m_ 36.3 ± 3.6 µM). For the co-substrate NADPH coAaCYP98 + CPR displayed a *K*_m_ value of 53.2 ± 4.0 µM. In our hands, no saturation could be achieved up to 1 mM *p*-coumaroylshikimic acid and thus a *K*_*m*_ value for this substrate could not be determined. This observation indicates a rather high *K*_*m*_ value and thus a low affinity towards this substrate.

**Table 2:** Kinetic values of AaCYP98 (in combination with CPR from *Coleus blumei*) for *p*-coumaroyl-3-hydroxyanthranilic acid, *p*-coumaroylanthranilic acid, *p*-coumaroyltyramine and NADPH

*K*_m_ value for	With	MM: *K*_m_ [µM]	HW: *K*_m_ [µM]
*p*-Coumaroyl-3-hydroxyanthranilic acid	NADPH	9.3 ± 0.7	7.2 ± 0.9
*p*-Coumaroylanthranilic acid	NADPH	6.4 ± 0.8	5.5 ± 1.6
*p*-Coumaroyltyramine	NADPH	36.3 ± 3.6	34.3 ± 0.7
NADPH	*p*-Coumaroylanthranilic acid	53.2 ± 4.0	50.6 ± 3.4

Values were calculated with the help of Michaelis–Menten (MM) and Hanes–Woolf plots (HW). Mean values of at least 9 determinations ± standard error

## Discussion

One of our main interests in looking into the biosynthesis of hydroxycinnamic acid derivatives, especially RA, in *Anthoceros agrestis* was to investigate whether the biosynthesis of RA and the genes lying behind this pathway are evolutionary old traits that have been passed on to the seed plant families or whether this biosynthetic pathway has evolved several times independently. Recent investigations of members of the Lamiaceae and the Boraginaceae indicate that hydroxycinnamoyltransferases catalyzing the transesterification of a hydroxycinnamoyl-CoA and a hydroxyphenyllactic acid moiety have evolved independently in these two plant families (Levsh et al. 2019). The hydroxycinnamoyltransferase from *Phacelia campanularia* (Boraginaceae) proved to be more closely related to spermidine hydroxycinnamoyltransferases. Furthermore, two different CYP98 proteins (CYP98A112, CYP98A113) were found to be involved in finalizing the substitution patterns of the aromatic rings (Levsh et al. 2019) while only one (CYP98A14) is known to date from *Coleus blumei* as a member of the Lamiaceae (Eberle et al. 2009). Both enzyme families, BAHD acyltransferases and CYP98, have widely expanded at different time points during evolution (Tuominen et al. 2011; Alber et al. 2019). While only one candidate sequence for CYP98 has been identified in the genome database of *Anthoceros agrestis* and other early diverged plant groups as well as gymnosperms, already multiple gene candidates for acyltransferases of the BAHD superfamily are present in these plant taxa.

In search for the enzyme catalyzing the formation of RA or one of its less hydroxylated precursors in the hornwort *Anthoceros agrestis*, we searched the genome database of the closely related species *Anthoceros punctatus* (Kelly, personal communication) and came upon the partial sequence of scaffold 3426 with the highest identity to RAS from *Melissa officinalis* (Weitzel and Petersen 2011) among the BAHD-sequences in this hornwort. However, other less similar sequences of BAHD acyltransferases were found as well, none of which at the present time encoded a protein with RAS activity (own unpublished results). After publication of whole genome sequences of *Anthoceros agrestis* (Szövényi 2016; Carpenter et al. 2019; Leebens-Mack et al. 2019), we later used our full-length Aa*HCT6* sequence from *Anthoceros agrestis* suspension-cultured cells to search the 1kp database (https://db.cngb.org/onekp/) and identified scaffolds 2004822, 2004823 and 2050582 (with identical amino acid sequences) that displayed an identity of 48% to RAS from *Melissa officinalis* and 100% to our AaHCT6 amino acid sequence. The identities to other scaffolds ranged between 30 and 40%. For this reason, we directed our interest primarily towards Aa*HCT6* (corresponding to scaffold 3426 from *A. punctatus*) as the gene most similar to RAS from Lamiaceae. The encoded protein, however, was not active with 4-hydroxyphenyllactic acid, the substrate involved in RA biosynthesis, but accepted shikimic acid as acceptor substrate and—to a lesser extent—3-hydroxyanthranilic acid and 2,3-dihydroxybenzoic acid, but not anthranilic acid. 3-Hydroxyanthranilic acid and 2,3-dihydroxybenzoic acid have previously been shown to be substrates for hydroxycinnamoyl-CoA:shikimic acid hydroxycinnamoyltransferase from *Coleus blumei* (CbHST) (Sander and Petersen 2011) and the former was also converted by RAS from *Lavandula angustifolia* (Landmann et al. 2011).

Kinetic characterization showed that *p*-coumaroyl-CoA was preferred as hydroxycinnamoyl donor over caffeoyl-CoA (except with 2,3-dihydroxybenzoic acid), while the enzyme displayed a higher affinity towards shikimic acid compared to 3-hydroxyanthranilic and 2,3-dihydroxybenzoic acids, the latter two probably do not serve as substrates *in planta*. Reactions with shikimic and 2,3-dihydroxybenzoic acids led to ester formation while the reaction with 3-hydroxyanthranilic acid has been shown to result in an amide (Suppl. Table S2). It must be pointed out here, that we did not yet determine which of the hydroxyl groups of 2,3-dihydroxybenzoic acid has been used for ester formation. Substrate promiscuity had already been shown for a number of other hydroxycinnamoyltransferases from plants forming esters as well as amides (Sander and Petersen 2011; Landmann et al. 2011; Eudes et al. 2016; Kriegshauser et al. 2021). A hydroxycinnamoyltransferase acting on anthranilic acid as substrate has been described from carnation (*Dianthus caryophyllus*; Caryophyllaceae), while hydroxycinnamoyltransferases from *Avena sativa* transfer the hydroxycinnamoyl moiety to 5-hydroxyanthranilic acid in avenanthramide biosynthesis (Yang et al. 1997, 2004). Hydroxycinnamoyltransferases from the moss *Physcomitrium patens* and the lycophyte *Selaginalla moellendorffii* accepted 3-hydroxy- and 5-hydroxyanthranilic acids besides shikimic acid and a variety of other phenolic compounds (Eudes et al. 2016). Liverwort hydroxycinnamoyltransferases from *Marchantia paleacea* and *Plagiochasma appendiculatum* were shown to be active with shikimic and quinic acids, however, other substrates have not been tested (Wu et al. 2018).

In the phylogenetic tree (Suppl. Fig. S2), the sequences encoding the above-mentioned enzymes from bryophytes and lycophytes group closely together, while the *Avena sativa* hydroxycinnamoyltransferases group together with other sequences from monocotyledonous angiosperms and the *Dianthus caryophyllus* enzyme is in a mixed group of hydroxycinnamoyltransferases far apart. This supports the hypothesis that ester as well as amide formation by BAHD hydroxycinnamoyltransferases has evolved independently several times and may be an intrinsic capability of this enzyme family. Overall, the substrate specificities of hydroxycinnamoyltransferases from early diverged plants do not seem to differ substantially from those of seed plants, since all accept shikimic acid for ester formation as well as various other substrates, some of them leading also to the synthesis of hydroxycinnamoyl amides. This has recently been shown by Kriegshauser et al. (2021) who demonstrated that bryophyte hydroxycinnamoyltransferases from *Marchantia polymorpha* and *Physcomitrium patens* could readily complement an *Arabidopsis thaliana* mutant defective in the hydroxycinnamoyltransferase involved in monolignol formation. The *Physcomitrium patens* hydroxycinnamoyltransferase analyzed by Kriegshauser et al. (2021) finally turned out to prefer shikimic acid as acceptor substrate although previously a threonic acid accepting enzyme had been expected deduced from data from previous studies (Renault et al. 2017). Interestingly, feruloyl-CoA was described as best donor substrate for the *Physcomitrium patens* hydroxycinnamoyltransferase, although feruloylshikimic acid (as well as caffeoylshikimic acid) has not been identified as metabolite in plant extracts from *P. patens* in contrast to *p*-coumaroylshikimic acid (Kriegshauser et al. 2021).

The *meta*-hydroxylation of *p*-coumaric acid esters and amides to the respective caffeic acid derivatives is generally assigned to members of the CYP98 family; free *p*-coumaric acid itself is no or only a very poor substrate. The substrate specificity of CYP98 proteins from different plant species can vary considerably. As stated by Alber et al. (2019), the number of CYP98 genes and isoforms is restricted to one in bryophytes, lycophytes, monilophytes and gymnosperms but duplication and neo-functionalization events in angiosperms have increased the number of CYP98 candidates. Among the CYP98 enzymes, substrate promiscuity is a common feature. However, CYP98s from early diverged plants may display different substrate preferences than those from gymno- and angiosperms. While the latter preferably use *p*-coumaroylshikimic acid as substrate but can also hydroxylate *p*-coumaroylquinic acid to varying extents, the single *Physcomitrium patens* CYP98 was reported to accept *p*-coumaroylthreonic acid and *p*-coumaroylanthranilic acid (Renault et al. 2017), but also *p*-coumaroylshikimic acid (Alber et al. 2019). As reported by Alber et al. (2019), phenol amides (e.g., with anthranilic acid and spermidine) were preferred over phenol esters. However, this view is changing due to the recent publication of Kriegshauser et al. (2021) who postulated that *p*-coumaroylthreonic acid is not a native substrate of CYP98 from *Physcomitrium patens*. Since only one CYP98 sequence is present in *Anthoceros agrestis*, we assumed this to be directly involved in RA biosynthesis. After identifying the respective full-length sequence, first attempts with expression in the frequently used expression system *Saccharomyces cerevisiae* were not successful. We speculated that either the different GC-content of yeast (average 39% in yeast; Nakase and Komagata 1971) or *Antho-ceros agrestis* CYP98 (62%) and/or the differing codon usage (codon usage database: www.kazusa.or.jp) might be the reason. Codon optimization of the hornwort gene was therefore the matter of choice and besides adapting the codons for *Saccharomyces cerevisiae*, the GC content was lowered to 36%. Active protein was synthesized by transformed yeast to a low extent using the electron transfer from yeast CPR. Combination of AaCYP98 with CPR from *Coleus blumei* (Eberle et al. 2009), however, strongly increased the hydroxylation activities and therefore this system was used for further characterization. Using a number of putative substrates, substrate promiscuity with preference for hydroxycinnamoyl amides was found for CYP98A147 from the hornwort *Anthoceros agrestis*. Similar affinities were found for *p*-coumaroylanthranilic acid (*K*_m_ 6.4 ± 0.8 µM) and *p*-coumaroyl-3-hydroxyanthranilic acid (*K*_m_ 9.3 ± 0.7 µM) and lower affinity for *p*-coumaroyltyramine (*K*_m_ 36.3 ± 3.6 µM), all of them *p*-coumaroyl amides. The *p*-coumaroyl esters with shikimic and 4-hydroxyphenyllactic acids were accepted as well, but only resulted in rather low product formation (Table 2 and Suppl. Fig. S12). Even at 1 mM *p*-coumaroylshikimic acid, the hydroxylase was not saturated pointing at a rather low affinity towards this substrate. On the other hand, AaHCT6 catalyzed preferentially the formation of *p*-coumaroylshikimic acid, followed by *p*-coumaryol-3-hydroxyanthranilic acid, while *p*-coumaroylanthranilic acid (one of the preferred substrates of CYP98A147) was not formed. Similarly, Eudes et al. (2016) reported that a *Physcomitrium patens* hydroxy-cinnamoyltransferase did not catalyze *p*-coumaroylanthranilic acid formation but only accepted 3- and 5-hydroxyanthranilic acid as acceptors. Threonic acid, esterified to *p*-coumaroylthreonic acids, which were postulated to play an essential role in the formation of phenol-enriched cuticles in *Physcomitrium patens* (Renault et al. 2017), was not a substrate for AaHCT6 and *p*-coumaroylthreonic acid was not converted by AaCYP98.

Interestingly, the hydroxylation of the RA precursor *p*-coumaroyl-4’-hydroxyphenyllactic acid by AaCYP98 was very low, although *Anthoceros agrestis* accumulates substantial amounts of RA (Vogelsang et al. 2006). In *Coleus blumei*, CYP98A14 only accepts *p*-coumaroyl-4′-hydroxyphenyllactic acid, *p*-coumaroyl-3′,4′-dihydroxyphenyllactic acid and caffeoyl-4′-hydroxyphenyllactic acid while *p*-coumaroylshikimic acid and *p*-coumaroylquinic acid were not converted (Eberle et al. 2009). The only identified CYP98 in *Anthoceros agrestis*, namely CYP98A147, might therefore not be directly involved in RA biosynthesis. An alternative way towards RA might follow a pathway including an early hydroxylation to the caffeic acid substitution pattern and the transfer of this caffeoyl moiety from an activated precursor to 4-hydroxyphenyl- or 3,4-dihydroxyphenyllactic acid. Recently, the 3-hydroxylation of 4-coumaric acid to caffeic acid by a bifunctional peroxidase that oxidizes both ascorbic acid and 4-coumaric acid and its impact on monolignol formation has been described from grasses, *Medicago truncatula* and *Arabidopsis thaliana* (Barros et al. 2019).

Recently, Kriegshauser et al. (2021) performed metabolic analyses of plant material of different bryophytes and detected *p*-coumaroyl-5-*O*-shikimic acid as well as caffeoyl-5-*O*-shikimic acid in extracts of *Marchantia polymorpha* and *Anthoceros agrestis.* Only the *p*-coumaroyl derivative was found in gametophore extracts of *Physcomitrium patens*, although CYP98 expression—presumably leading to caffeoylshikimic acid—had been shown in *P. patens* at this developmental stage (Renault et al. 2017). This shows the presence of hydroxycinnamoylshikimic acid esters in all bryophyte groups. At the same time, the respective threonic acid esters have only been detected in *Physcomitrium patens* (Kriegshauser et al. 2021). Earlier reports by Trenn-heuser (1992)—a very detailed phytochemical investigation—and Takeda et al. (1990b) do not mention the presence of *p*-coumaroyl- or caffeoylshikimic acids in *Anthoceros*. However, the presence of hydroxycinnamoylshikimic acid esters in *Anthoceros agrestis* is in line with our observation that shikimic acid is the best substrate for AaHCT6. To our knowledge, Renault et al. (2017) did not test PpCYP98 with *p*-coumaroylshikimic acid. The only mediocre acceptance of *p*-coumaroylshikimic acid as substrate for AaCYP98 may be due to the test conditions although our assays with *p*-coumaroyl(hydroxy)anthranilic acids performed under the same conditions have been successful.

The presented results leave a final conclusion towards the biosynthesis of caffeoyl esters, especially rosmarinic acid, in the hornwort *Anthoceros agrestis* open and strengthen the probability that the formation of RA might follow a different way in this hornwort. This is especially the case for hydroxycinnamoyltransferases, because different classes of enzymes might catalyze the formation of hydroxycinnamic acid esters (see review by Petersen 2016). Since only a single CYP98 member has been found in hornworts, we either did not meet the requirements of this enzyme in vitro or the hydroxylation may take place prior to esterification. All in all, the formation of phenolic compounds in *Anthoceros agrestis* remains a field of research still to be conquered.

### *Author contribution statement*

LE, JW, EB and MP performed experiments and wrote the article. MP finalized the data and the text. All authors read and approved the manuscript.

## Supplementary Information

Below is the link to the electronic supplementary material.

Supplementary file1 (PDF 3258 KB)

## Data Availability

Sequence data were deposited in GenBank (https://https.ncbi.nlm.nih.gov/genbank/) under the accession numbers MW248389 and MT119883. The datasets generated and/or analyzed during the current study are available from the corresponding author on reasonable request.

## Abbreviations

BAHD: Acyltransferase family named according to the first four identified enzymes
CPR: NADPH:cytochrome P450 reductase
HCT: Hydroxycinnamoyltransferase
HST: Hydroxycinnamoyl-CoA:shikimic acid hydroxycinnamoyltransferase
RA: Rosmarinic acid
RAS: Rosmarinic acid synthase = hydroxycinnamoyl-CoA:hydroxyphenyllactic acid hydroxycinnamoyltransferase
