# Supplementary material for: Hydroxycinnamoyltransferase and CYP98 in phenolic metabolism in the rosmarinic acid-producing hornwort Anthoceros agrestis
Source: Planta. 2022 Mar 2;255(4):75. doi: 10.1007/s00425-022-03856-9 (PMC8891189; doi:10.1007/s00425-022-03856-9)
Supplement: Supplementary file 1 — Supplementary file1 (PDF 3258 KB) [file 425_2022_3856_MOESM1_ESM.pdf]

Supplementary Material for

Lucien Ernst, Julia Wohl, Elke Bauerbach, Maike Petersen

Hydroxycinnamoyltransferase and CYP98 in phenolic metabolism in the rosmarinic acid-producing hornwort *Anthoceros agrestis*

Supplementary Tables

Suppl. Table S1 List of PCR primers. Binding sequence underlined, restriction sites marked red

Primer name	Sequence
Primers for amplification of *CYP98*	
Aa26091_f	ATGGTGCACAAGATCCACC
Aa26091_r	GACGTTTCTGGCATACAAGG
Aa26091_5´R	GATTACGCCAAGCTTCCTTCCACACAGCAGGATCCCTGGC
Aa26091_3´R	GATTACGCCAAGCTTCGTTGACGCTCTGCTAGGGCTGC
Aa26091_fl_f	GCATGAATTCATGGCGGAGCTGGTGGTGA
Aa26091_fl_r	GCATGCGGCCGCGACGTTTCTGGCATACAAGGCC
MCSI_f	CTGAAAGTTCCAAAGAGAAG
MCSI_r	TTTCTGGCAAGGTAGACAAG
MCSII_f	GCCTTATTTCTGGGGTAATTAATCAGCG
MCSII_r	GTCCCAAAACCTTCTCAAGCAAGG
Primers for amplification of *HCT*	
Ap3426_f	GAGGCGGACGTGAGCATCG
Ap3426_r	TCAAATGTCATTCACAAGCTTCCTGAAC
Aa3426_5'R	CCCCCATCTTCAGCACAGCCTCC
Aa3426_3'R	AGCACGTCGAGTACCAGACGCCC
Aa3426_VL-f	AAACATATGAAGGTGAGCATCAAGAAGGAAACAATGG
Aa3426_VL-r	TTGGATCCTCAAATGTCATTCACAAGCTTCCTGAACTCAG

Suppl. Table S2 NMR data of chemically synthesized hydroxycinnamic acid derivatives. All NMR spectra were obtained in deuterated DMSO with a Jeol ECZ400S spectrometer and processed with Delta 5.3.1. In the NOESY experiment for *p*-coumaroyl-3-hydroxyanthranilic acid, the relaxation delay was set to 1.5 s and the mixing time was 1 s. The HMBC experiment was optimized for ^{1}J coupling constants between 128 and 163 Hz and long range couplings of 8 Hz. Assignment of the ^{1}H and ^{13}C signals for *p*-coumaroyl-3-hydroxyanthranilic acid was supported by APT and HMQC spectra

**Compound**	**NMR data**
p-Coumaroyl-tyramine	^{1}H NMR (400 MHz, DMSO-*d6*): *δ* [ppm] = 9.79 (s, 1H, OH), 9.13 (s, 1H, OH), 7.97 (t, *J* = 5.5 Hz, 1H, NH), 7.37 (dd, *J* = 8.7, 2.1 Hz, 2H, H_{Ar}), 7.30 (d, *J* = 15.8 Hz, 1H, H_{Olefin}), 7.05 – 6.96 (m, 2H, H_{Ar}), 6.78 (dd, *J* = 8.6, 2.1 Hz, 2H, H_{Ar}), 6.70 – 6.64 (m, 2H, H_{Ar}), 6.39 (d, *J* = 15.7 Hz, 1H, H_{Olefin}), 3.31 (s, 2H, CH2), 2.64 (t, *J* = 7.3 Hz, 2H, $C_{Ar}CH_2$)
p-Coumaroyl-anthranilic acid	^{1}H NMR (400 MHz, DMSO-*d6*): *δ* [ppm] = 13.57 (s, 1H, COOH), 11.26 (s, 1H, NH), 9.97 (s, 1H, OH), 8.62 (d, *J* = 8.4 Hz, 1H, H_{Ar}), 8.00 (dd, *J* = 7.9, 1.6 Hz, 1H, H_{Ar}), 7.58 (m, 4H, H_{Ar} und H_{Olefin}), 7.16 (dd, *J* = 10.7, 4.5 Hz, 1H, H_{Ar}), 6.82 (d, *J* = 8.5 Hz, 2H, H_{Ar}), 6.63 (d, *J* = 15.6 Hz, 1H, H_{Olefin})
p-Coumaroyl-3-hydroxyanthranilic acid	^{1}H NMR (400 MHz, DMSO-d_6): δ (ppm) = 6.75 (d, J = 15.6 H, 1H; 2´), 6.80-6.82 (part of an AA´XX´ system), 2H; H-3´´, H-5´´), 7.07 (dd, J = 8.1 Hz, 1.7 Hz, 1H; H-4), 7.12 (*pseudo*-t, J ≈ 7.8 Hz, 1H; H-5), 7.28 (dd, J = 7.4 Hz, 1.7 Hz, 1H; H-6), 7.45-7.48 (m, 3 H; H-3´, H-2´´, H-6´´), 9.90 (s, 1H; OH-4´´), 9.94 (s, 1H; OH-3), 10.21 (bs, 1H; NH), 12.71 (bs, 1H; COOH)
	^{13}C (100 MHz, DMSO-d_6): δ (ppm) = 115.8 (C-3´´, C-5´´), 117.9 (C-2´), 120.2 (C-4), 120.9 (C-6), 125.3 (C-2), 125.6 (C-5), 125.6 (C-1´´), 127.4 (C-1), 129.6 (C-2´´, C-6´´), 140.9 (C-3´), 151.0 (C-3), 159.2 (C-4´´), 165.0 (C-1´), 168.2 (COOH).

To distinguish between the amide and the ester, NOESY and HMBC NMR spectra were recorded. The observed correlations in the NOESY NMR spectrum between two protons on heteroatoms and different aromatic protons can only occur in the amide. Coupling between both oxygen-bound aromatic carbons and protons on heteroatoms in the HMBC spectrum

additionally points to the amide. Furthermore, the neighbouring C-4 could not show any coupling to protons on heteroatoms in the ester

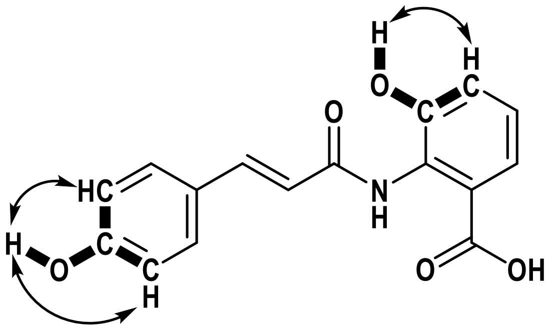

Key correlations in the NOESY spectrum (arrows) and key couplings in the HMBC spectrum (bold bonds)

Suppl. Table S3 Substrate variation for the determination of kinetic constants for AaHCT6

**Varied substrate**		**Constant substrate**	
3-Hydroxyanthranilic acid	0-4 mM	*p*-Coumaroyl-CoA	0.1 mM
		Caffeoyl-CoA	0.2 mM
p-Coumaroyl-CoA	0-0.14 mM	3-Hydroxyanthranilic acid	0.4 mM
Caffeoyl-CoA	0-0.8 mM		4 mM
Shikimic acid	0-6 mM	*p*-Coumaroyl-CoA	0.1 mM
	0-20 mM	Caffeoyl-CoA	0.1 mM
p-Coumaroyl-CoA	0-0.2 mM	Shikimic acid	0.4 mM
Caffeoyl-CoA	0-0.8 mM		0.4 mM
2,3-Dihydroxybenzoic acid	0-8 mM	*p*-Coumaroyl-CoA	0.1 mM
		Caffeoyl-CoA	0.1 mM
p-Coumaroyl-CoA	0-0.2 mM	2,3-Dihydroxybenzoic acid	0.4 mM
Caffeoyl-CoA	0-0.8 mM		

Suppl. Table S4 Compounds that did not result in product formation with AaHCT6 (in alphabetical order)

1-Butanol	4-Hydroxyphenyllactic acid	Putrescine
1-Phenylethanol	4-Phenyl-1-butanol	Quinic acid
1-Propanol	Agmatine	Salicylic acid
2-(4-Hydroxyphenyl)-1-ethanol	Anthranilic acid	Serotonin
2,4-Dihydroxybenzoic acid	Benzoic acid	Spermidine
2,5-Dihydroxybenzoic acid	Dihydroxyphenylalanine (L-, DL-)	Spermine
2-Phenethylamine	Dopamine	Tartaric acid (D-, L-, m-)
2-Phenylethanol	Ethanol	Threonic acid (D-, L-)
3-(4-Hydroxyphenyl)-1-propanol	Glucaric acid	Tryptamine
3,4-Dihydroxybenzoic acid	Glutamic acid	Tryptophan (D-, L-)
3-Aminobenzoic acid	Isopropanol	Tyramine
3-Aminosalicylic acid	Malic acid (D-, L-, DL-)	Tyrosine (D-, L-)
3-Hydroxybenzoic acid	Methanol	Vanillic acid
3-Phenyl-1-propanol	Mucic acid	
4-Hydroxybenzoic acid	Phenylalanine (D-, L-)	

Supplementary Figures

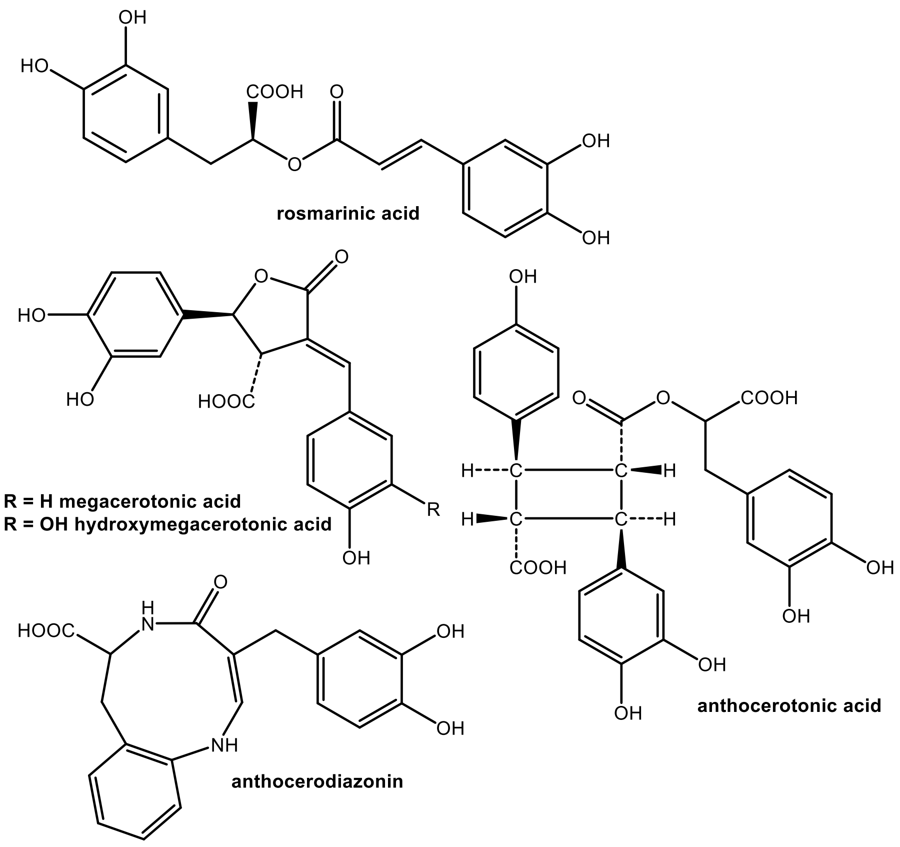

Suppl. Fig. S1 Structures of some hydroxycinnamic acid derivatives in hornworts

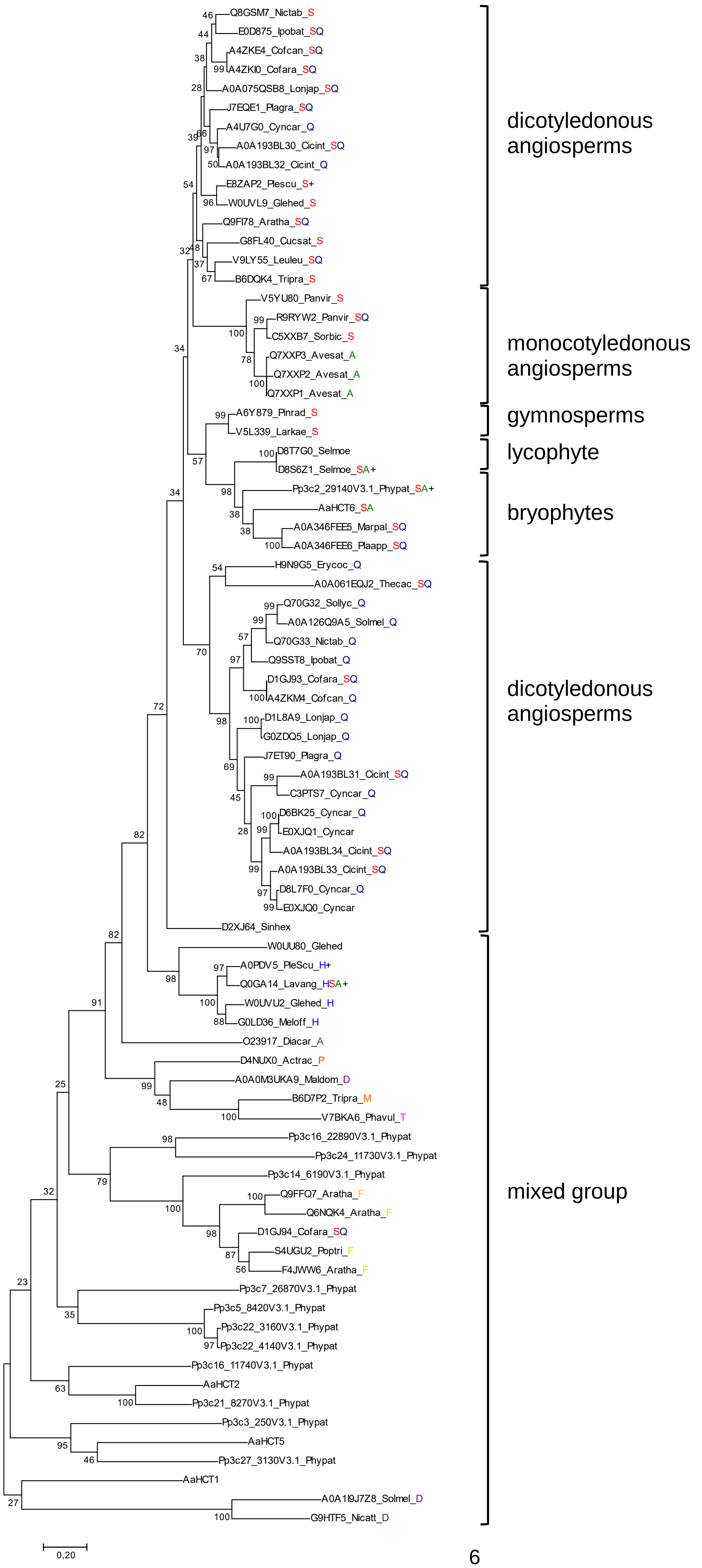
Q8GSM7_Nictab_S
E0D875_Ipobat_SQ
A4ZKE4_Cofcan_SQ
A4ZKI0_Cofara_SQ
A0A075QSB8_Lonjap_SQ
J7EQE1_Plagra_SQ
A4U7G0_Cyncar_Q
A0A193BL30_Cicint_SQ
A0A193BL32_Cicint_Q
E8ZAP2_Plescu_S+
W0UVL9_Glehed_S
Q9FI78_Aratha_SQ
G8FL40_Cucsat_S
V9LY55_Leuleu_SQ
B6DQK4_Tripra_S
V5YU80_Panvir_S
R9RYW2_Panvir_SQ
C5XXB7_Sorbic_S
Q7XXP3_Avesat_A
Q7XXP2_Avesat_A
Q7XXP1_Avesat_A
A6Y879_Pinrad_S
V5L339_Larkae_S
D8T7G0_Selmoe
D8S6Z1_Selmoe_SA+
Pp3c2_29140V3.1_Phypat_SA+
AaHCT6_SA
A0A346FEE5_Marpal_SQ
A0A346FEE6_Plaapp_SQ
H9N9G5_Erycoc_Q
A0A061EQJ2_Thecac_SQ
Q70G32_Sollyc_Q
A0A126Q9A5_Solmel_Q
Q70G33_Nictab_Q
Q9SST8_Ipobat_Q
D1GJ93_Cofara_SQ
A4ZKM4_Cofcan_Q
D1L8A9_Lonjap_Q
G0ZDQ5_Lonjap_Q
J7ET90_Plagra_Q
A0A193BL31_Cicint_SQ
C3PTS7_Cyncar_Q
D6BK25_Cyncar_Q
E0XJQ1_Cyncar
A0A193BL34_Cicint_SQ
A0A193BL33_Cicint_SQ
D8L7F0_Cyncar_Q
E0XJQ0_Cyncar
D2XJ64_Sinhex
W0UU80_Glehed
A0PDV5_PleScu_H+
Q0GA14_Lavang_HSA+
W0UVU2_Glehed_H
G0LD36_Meloff_H
O23917_Diacar_A
D4NUX0_Actrac_P
A0A0M3UKA9_Maldom_D
B6D7P2_Tripra_M
V7BKA6_Phavul_T
Pp3c16_22890V3.1_Phypat
Pp3c24_11730V3.1_Phypat
Pp3c14_6190V3.1_Phypat
Q9FFQ7_Aratha_F
Q6NQK4_Aratha_F
D1GJ94_Cofara_SQ
S4UGU2_Poptri_F
F4JWW6_Aratha_F
Pp3c7_26870V3.1_Phypat
Pp3c5_8420V3.1_Phypat
Pp3c22_3160V3.1_Phypat
Pp3c22_4140V3.1_Phypat
Pp3c16_11740V3.1_Phypat
AaHCT2
Pp3c21_8270V3.1_Phypat
Pp3c3_250V3.1_Phypat
AaHCT5
Pp3c27_3130V3.1_Phypat
AaHCT1
A0A1I9J7Z8_Solmel_D
G9HTF5_Nicatt_D
0,20
dicotyledonous angiosperms
monocotyledonous angiosperms
gymnosperms
lycophyte
bryophytes
dicotyledonous angiosperms
mixed group

Suppl. Fig. S2 Molecular phylogenetic analysis of BAHD hydroxycinnamoyltransferase amino acid sequences by the Maximum Likelihood method of the MEGA7 software package. The evolutionary history was inferred by using the Maximum Likelihood method based on the JTT matrix-based model (Jones et al. 1992). The tree with the highest log likelihood (-22142,1698) is shown. The percentage of trees in which the associated taxa clustered together is shown next to the branches. Initial tree(s) for the heuristic search were obtained automatically by applying Neighbor-Join and BioNJ algorithms to a matrix of pairwise distances estimated using a JTT model, and then selecting the topology with superior log likelihood value. The tree is drawn to scale, with branch lengths measured in the number of substitutions per site. The analysis involved 80 amino acid sequences. All positions containing gaps and missing data were eliminated. There were a total of 279 positions in the final dataset. Evolutionary analyses were conducted in MEGA7 (Kumar et al. 2016). Aa – *Anthoceros agrestis*, Actrac – *Althaea racemosa*, Aratha – *Arabidopsis thaliana*, Avesat – *Avena sativa*, Cicint – *Cichorium intybus*, Cofara – *Coffea arabica*, Cofcan – *Coffea canephora*, Cucsat – *Cucumis sativus*, Cyncar – *Cynara cardunculus*, Diacar – *Dianthus caryophyllus*, Erycoc – *Erythroxylum coca*, Glehed – *Glechoma hederacea*, Ipobat – *Ipomoea batatas*, Larkae – *Larix kaempferi*, Lavang – *Lavandula angustifolia*, Leuleu – *Leucocephalum leucaenum*, Lonjap – *Lonicera japonica*, Maldom – *Malus domestica*, Marpal – *Marchantia palaeacea*, Meloff – *Melissa officinalis*, Nicatt – *Nicotiana attenuata*, Nictab – *Nicotiana tabacum*, Panvir – *Panicum virgatum*, Phavul – *Phaseolus vulgaris*, Phypat – *Physcomitrium patens*, Pinrad – *Pinus radiata*, Plaapp – *Plagiochasma appendiculatum*, Plagra – *Platycodon grandiflorum*, Plescu – *Plectranthus scutellarioides*, Poptri – *Populus trichocarpa*, Selmoe – *Selaginella moellendorffii*, Sinhex – *Sinopodophyllum hexandrum*, Sollyc – *Solanum lycopersicum*, Solmel – *Solanum melongena*, Sorbic – *Sorghum bicolor*, Thecac – *Theobroma cacao*, Tripra – *Trifolium pratense*. Accession numbers are given in the tree's branches. The letters following the species abbreviation indicate the acceptor substrates (colour-coded):

- A Anthranilic / Hydroxyanthranilic acid
- D Spermidine / Spermine
- F Fatty acids
- H 4-Hydroxyphenyllactic acid
- M Malic acid
- P Piscidic acid
- Q Quinic acid
- S Shikimic acid
- T Tetrahydroxyhexanedioic acid
- ☐ Other substrates

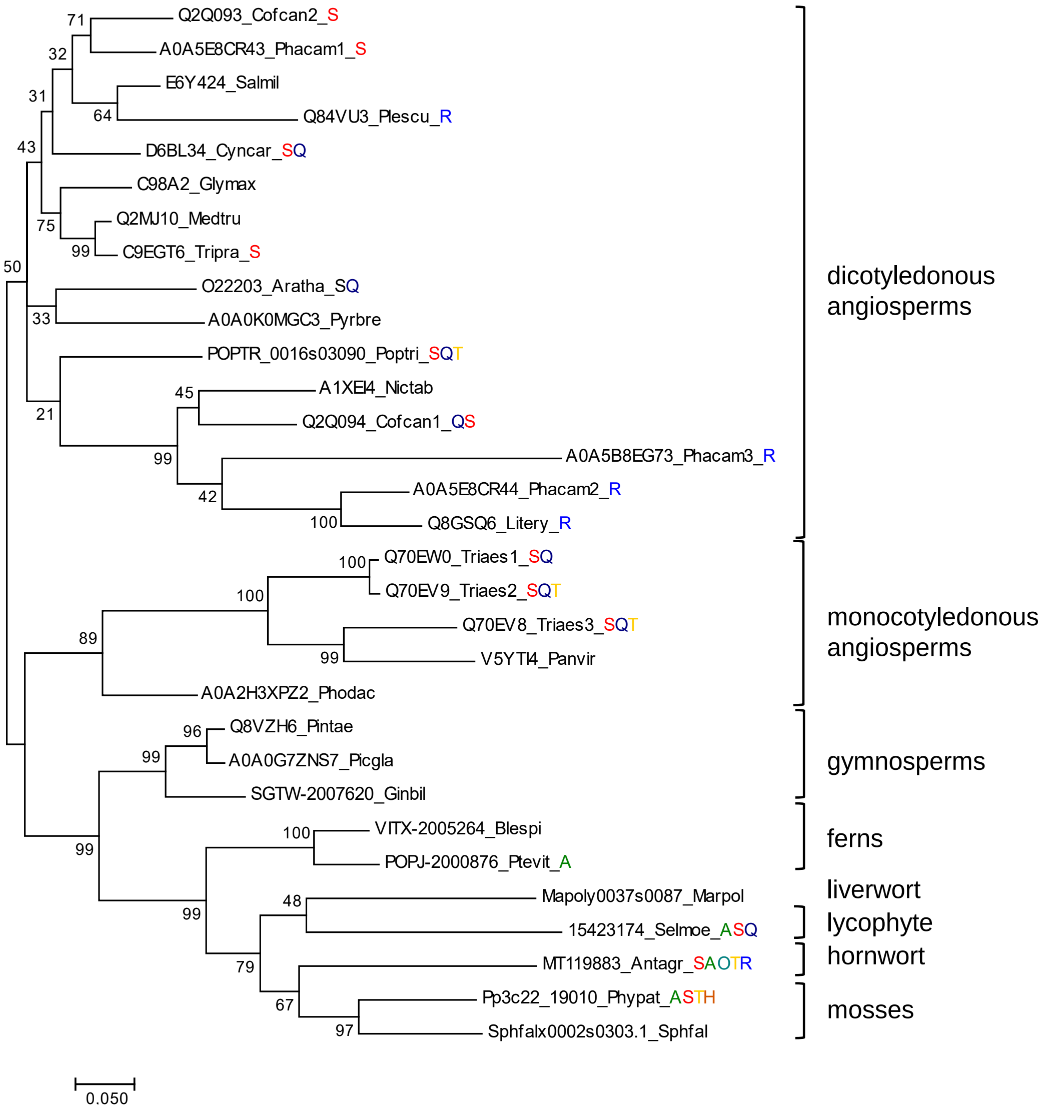

Suppl. Fig. S3 Molecular phylogenetic analysis of CYP98 amino acid sequences by the Maximum Likelihood method of the MEGA7 software package. The evolutionary history was inferred by using the Maximum Likelihood method based on the JTT matrix-based model (Jones et al. 1992). The tree with the highest log likelihood (-10289,9420) is shown. The percentage of trees in which the associated taxa clustered together is shown next to the branches. Initial tree(s) for the heuristic search were obtained automatically by applying Neighbor-Join and BioNJ algorithms to a matrix of pairwise distances estimated using a JTT model, and then selecting the topology with superior log likelihood value. The tree is drawn to scale, with branch lengths measured in the number of substitutions per site. The analysis involved 31 amino acid sequences. All positions containing gaps and missing data were eliminated. There were a total of 453 positions in the final dataset. Evolutionary analyses were conducted in MEGA7 (Kumar et al. 2016). Antagr – *Anthoceros agrestis*, Aratha – *Arabidopsis thaliana*, Blespi – *Blechnum spicant*, Cofcan - *Coffea canephora*, Cyncar – *Cynara*

cardunculus, Ginbil – *Ginkgo biloba*, Glymax – *Glycine max*, Litery – *Lithospermum erythrorhizon*, Marpol – *Marchantia polymorpha*, Medtru – *Medicago truncatula*, Nictab – *Nicotiana tabacum*, Panvir – *Panicum virgatum*, Phacam – *Phacelia campanularia*, Phodac – *Phoenix dactylifera*, Phypat – *Physcomitrium patens*, Picglau – *Picea glauca*, Pintae – *Pinus taeda*, Plescu – *Plectranthus scutellarioides*, Poptri – *Populus trichocarpa*, Ptevit – *Pteris vittata*, Pyrbre – *Pyrus x brettschneideri*, Salmil – *Salvia milthiorrhiza*, Selmoe – *Selaginella moellendorffii*, Sphfal – *Sphagnum fallax*, Triaes – *Triticum aestivum*, Tripra – *Trifolium pratense*. Accession numbers are given in the tree's branches. The letters following the species abbreviation indicate the substrates that were hydroxylated (colour-coded):

A *p*-Coumaroylanthranilic acid

H *p*-Coumaroyl-2-threonic acid

O *p*-Coumaroyl-3-hydroxyanthranilic acid

Q *p*-Coumaroylquinic acid

R *p*-Coumaroyl-4'-hydroxyphenyllactic acid and/or *p*-coumaroyl-3',4'-dihydroxyphenyllactic acid and/or caffeoyl-4'-hydroxyphenyllactic acid

S *p*-Coumaroylshikimic acid

T *p*-Coumaroyltyramine

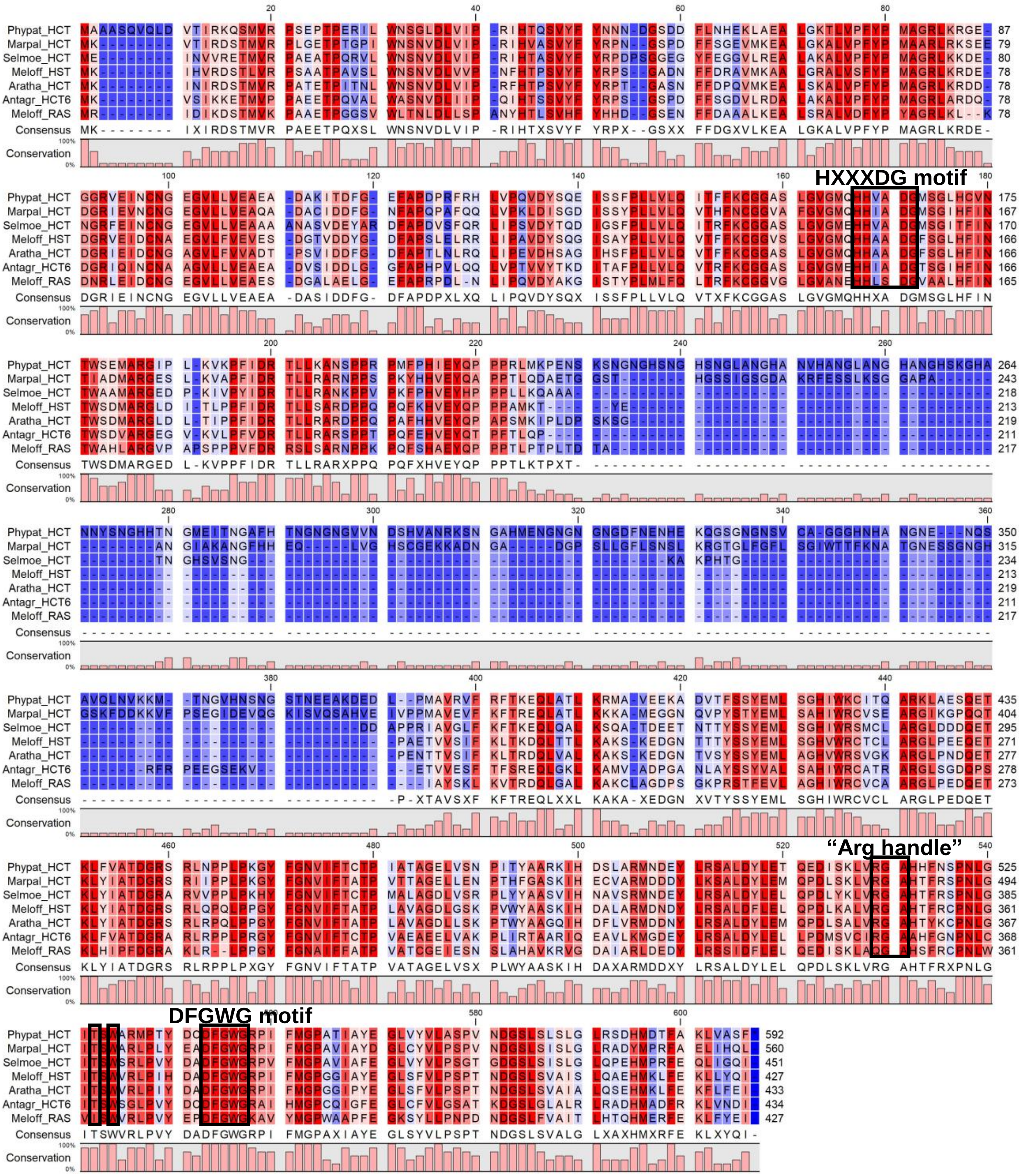

Suppl. Fig. S4 Alignment of hydroxycinnamoyltransferase (HCT) sequences from seed and non-seed plants. Phypat_HCT – *Physcomitrium patens* (XP_024361034), Marpal_HCT – *Marchantia palaeacea* (A0A346FEE5), Selmoe_HCT – *Selaginella moellendorfii* (D8S6Z1), Meloff_HST – hydroxycinnamoyl-CoA:shikimic acid hydroxycinnamoyltransferase from *Melissa officinalis* (own work), ArathaHCT – *Arabidopsis thaliana* (Q9FI78), Antagr_HCT6 – *Anthoceros agrestis* hydroxycinnamoyl-CoA:shikimic acid/3-hydroxyanthranilic acid hydroxy-cinnamoyltransferase (MW248389; this paper), Meloff_RAS – hydroxycinnamoyl-CoA:hydroxyphenyllactic acid hydroxycinnamoyltransferase from *Melissa officinalis* (G0LD36). Highly conserved amino acids are marked red, low conservation is marked blue. Typical conserved sequence motifs are marked by boxes

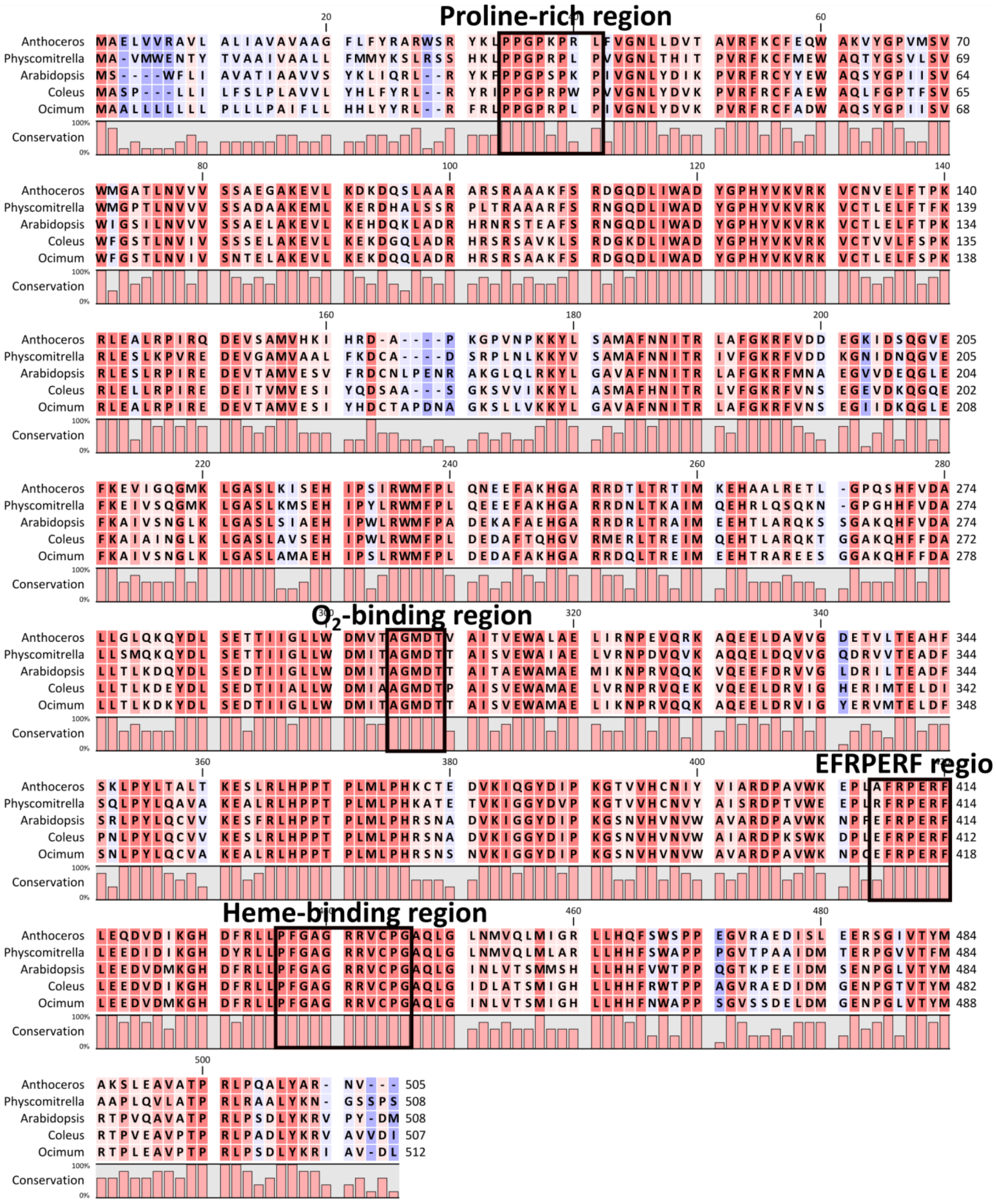

Suppl. Fig. S5 Alignment of CYP98 amino acid sequences from seed and non-seed plants. *Anthoceros agrestis* (this work), *Physcomitrium patens* (Pp3c22_19010), *Arabidopsis thaliana* (P850337), *Coleus blumei* (CAD20576) and *Ocimum basilicum* (AY082611). Highly conserved amino acids are marked red, low conservation is marked blue. Typical conserved sequence motifs are marked by boxes

Suppl. Fig. S6 Western blot of a SDS-PAGE of crude protein extracts from *E. coli* SoluBL21 transformed with AaHCT6 or with the empty vector pET-15b. The attached 6xHis-tag was visualized by mouse anti-6xHis-tag antibodies followed by goat anti-mouse antibodies conjugated to alkaline phosphatase and NBT/BCIP staining (The contrast was slightly improved.)

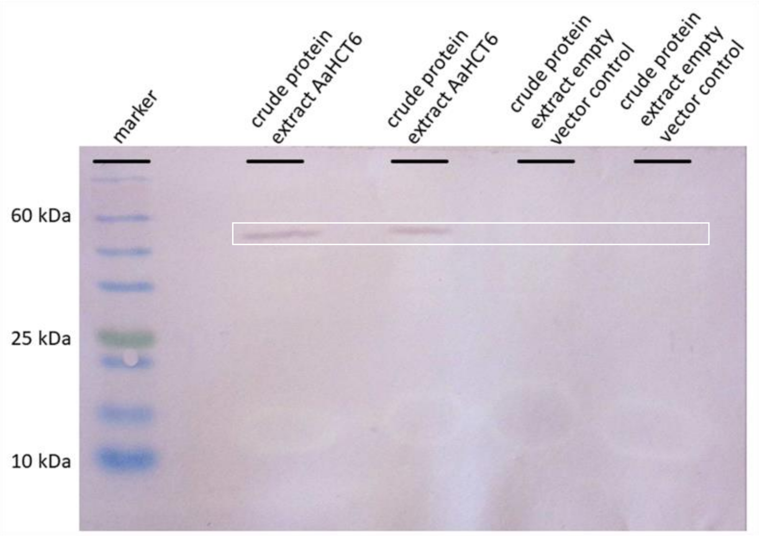

Suppl. Fig S7 Enzyme assay with AaHCT6 crude protein extract (black line) and protein extract from empty vector control (dashed grey line) using 4-coumaroyl-CoA and shikimic acid as substrates

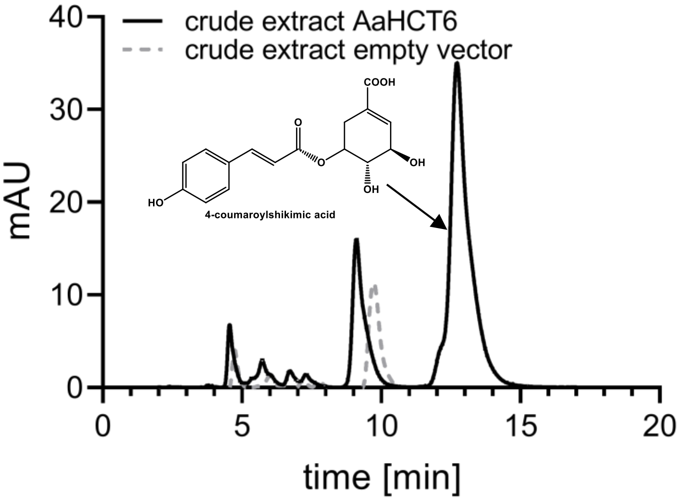

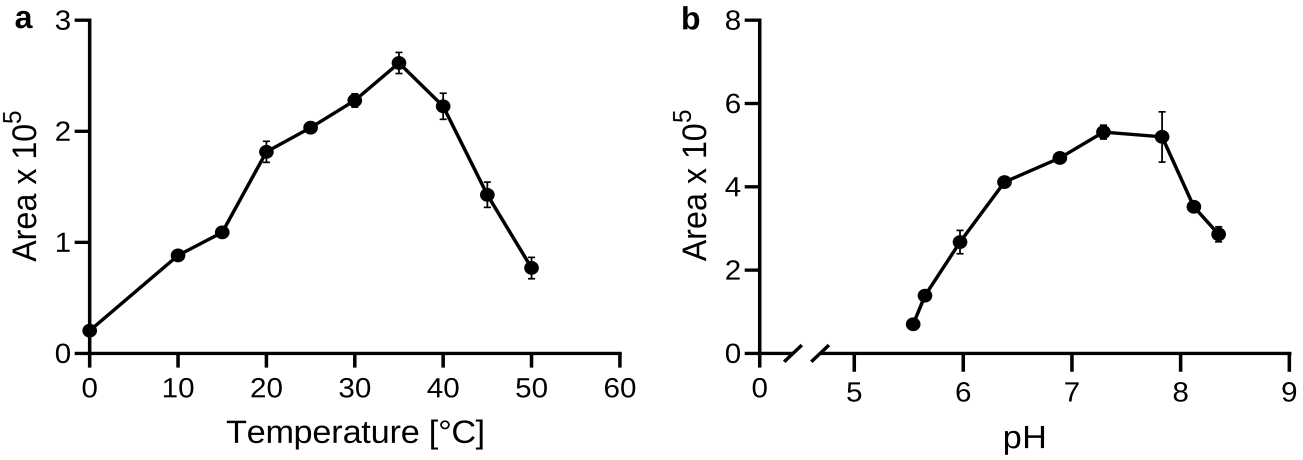

Suppl. Fig. S8 a Temperature optimum of AaHCT6 determined with caffeoyl-CoA and shikimic acid (n = 8 ± SE). **b** pH-optimum of AaHCT6 determined with caffeoyl-CoA and shikimic acid (n = 4 ± SE)

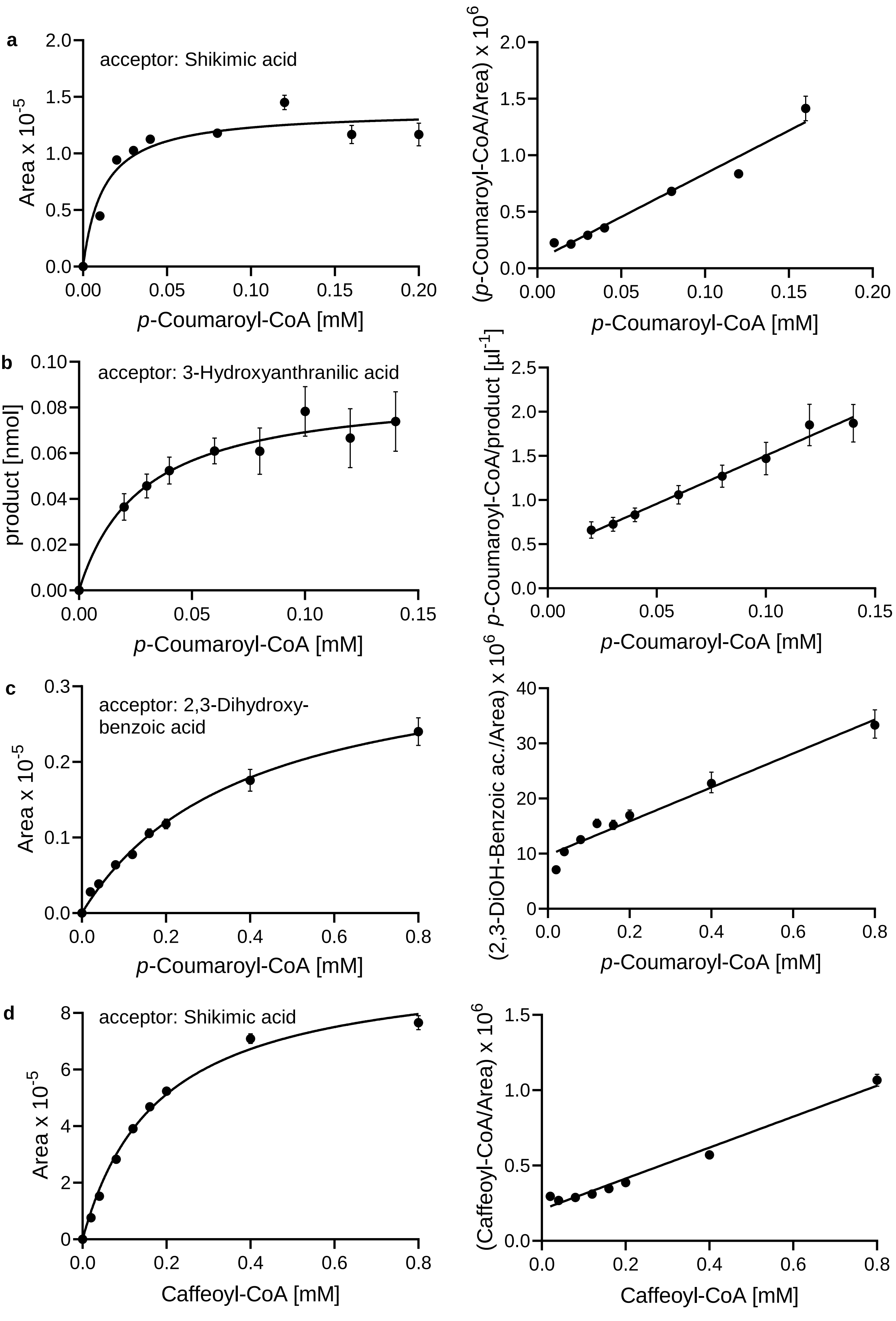
a
acceptor: Shikimic acid
Area x 10-5
p-Coumaroyl-CoA [mM]
(p-Coumaroyl-CoA/Area) x 10^6
p-Coumaroyl-CoA [mM]
b
acceptor: 3-Hydroxyanthranilic acid
product [nmol]
p-Coumaroyl-CoA [mM]
p-Coumaroyl-CoA/product [µl-1]
p-Coumaroyl-CoA [mM]
c
acceptor: 2,3-Dihydroxy-benzoic acid
Area x 10-5
p-Coumaroyl-CoA [mM]
(2,3-DiOH-Benzoic ac./Area) x 10^6
p-Coumaroyl-CoA [mM]
d
acceptor: Shikimic acid
Area x 10-5
Caffeoyl-CoA [mM]
(Caffeoyl-CoA/Area) x 10^6
Caffeoyl-CoA [mM]

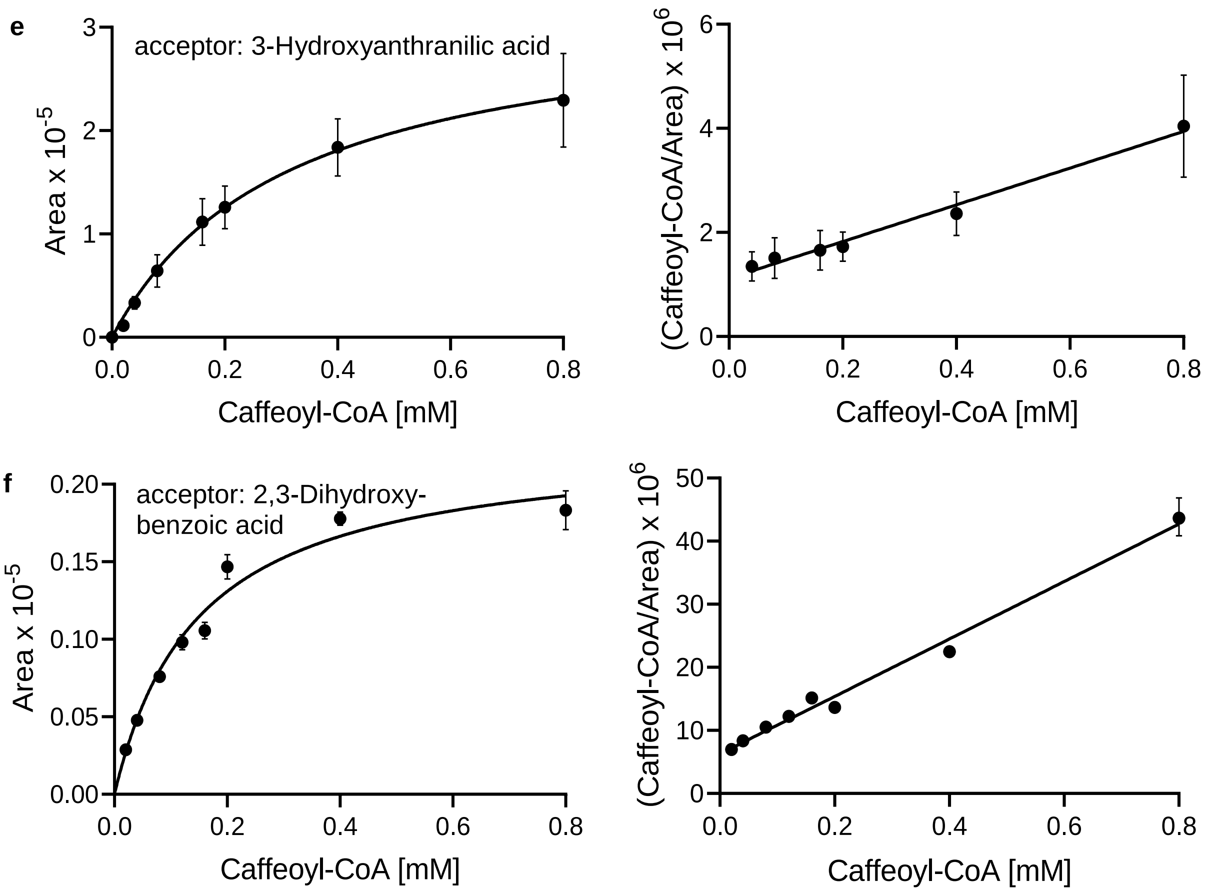

Suppl. Fig. S9 Determination of K_m-values for hydroxycinnamoyl-CoA derivatives with different acceptor substrates. Michaelis-Menten (left) and Hanes-Wolf (right) diagrams for AaHCT6. **a** *p*-Coumaroyl-CoA with shikimic acid (*n* = 12 ± SE). **b** *p*-Coumaroyl-CoA with 3-hydroxy-anthranilic acid (*n* = 9 ± SE). **c** *p*-Coumaroyl-CoA with 2,3-dihydroxybenzoic acid (*n* = 5 ± SE). **d** Caffeoyl-CoA with shikimic acid (*n* = 12 ± SE). **e** Caffeoyl-CoA with 3-hydroxyanthranilic acid (*n* = 12 ± SE). **f** Caffeoyl-CoA with 2,3-dihydroxybenzoic acid (*n* = 4 ± SE)

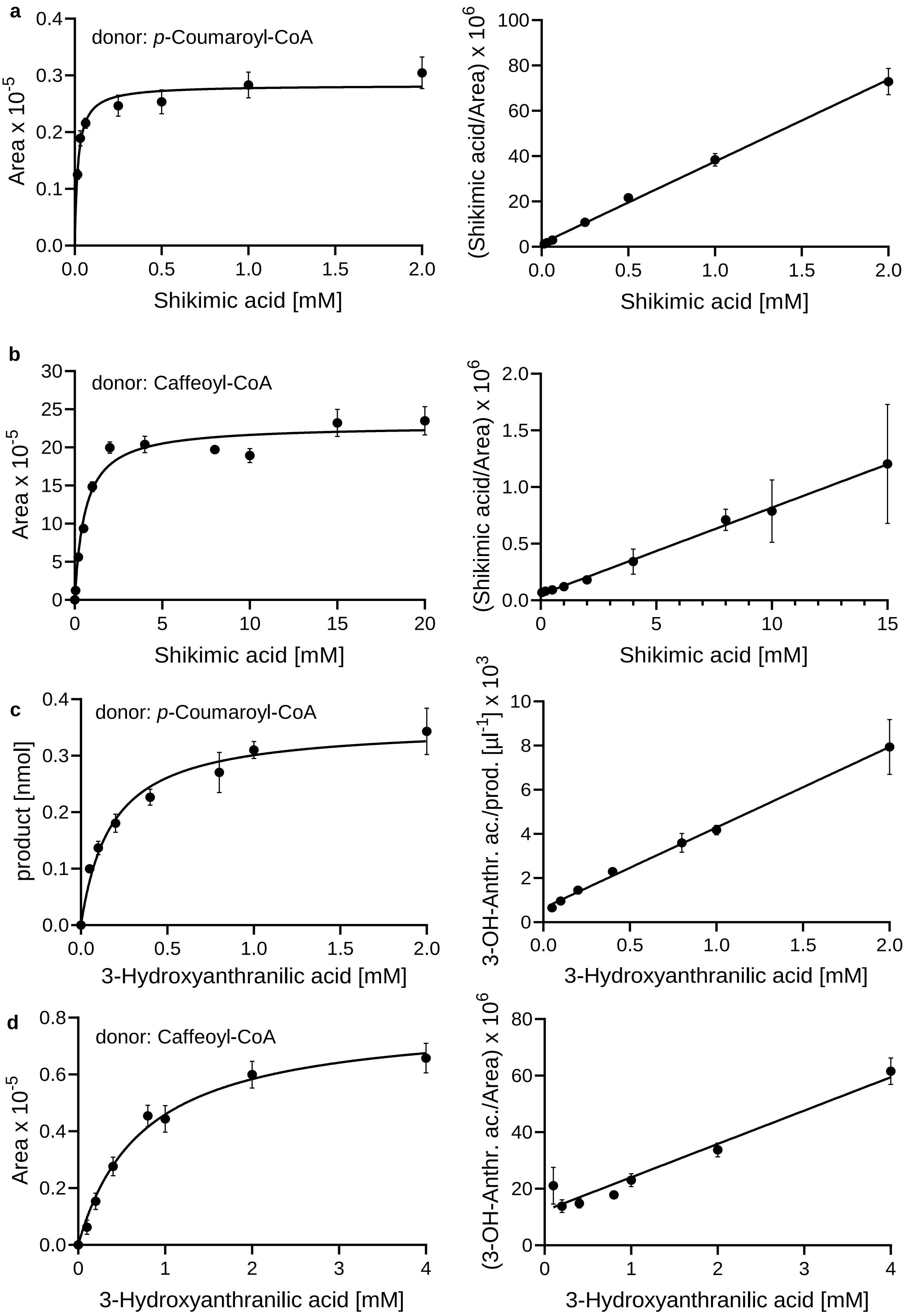
a
donor: p-Coumaroyl-CoA
Area x 10-5
0.0
0.1
0.2
0.3
0.4
0.0
0.5
1.0
1.5
2.0
Shikimic acid [mM]
(Shikimic acid/Area) x 106
0
20
40
60
80
100
Shikimic acid [mM]
b
donor: Caffeoyl-CoA
Area x 10-5
0
5
10
15
20
25
30
Shikimic acid [mM]
(Shikimic acid/Area) x 106
0.0
0.5
1.0
1.5
2.0
Shikimic acid [mM]
c
donor: p-Coumaroyl-CoA
product [nmol]
3-Hydroxyanthranilic acid [mM]
3-OH-Anthr. ac./prod. [µl-1] x 103
0
2
4
6
8
10
3-Hydroxyanthranilic acid [mM]
d
donor: Caffeoyl-CoA
Area x 10-5
0.0
0.2
0.4
0.6
0.8
0
1
2
3
4
3-Hydroxyanthranilic acid [mM]
(3-OH-Anthr. ac./Area) x 106
0
20
40
60
80
3-Hydroxyanthranilic acid [mM]

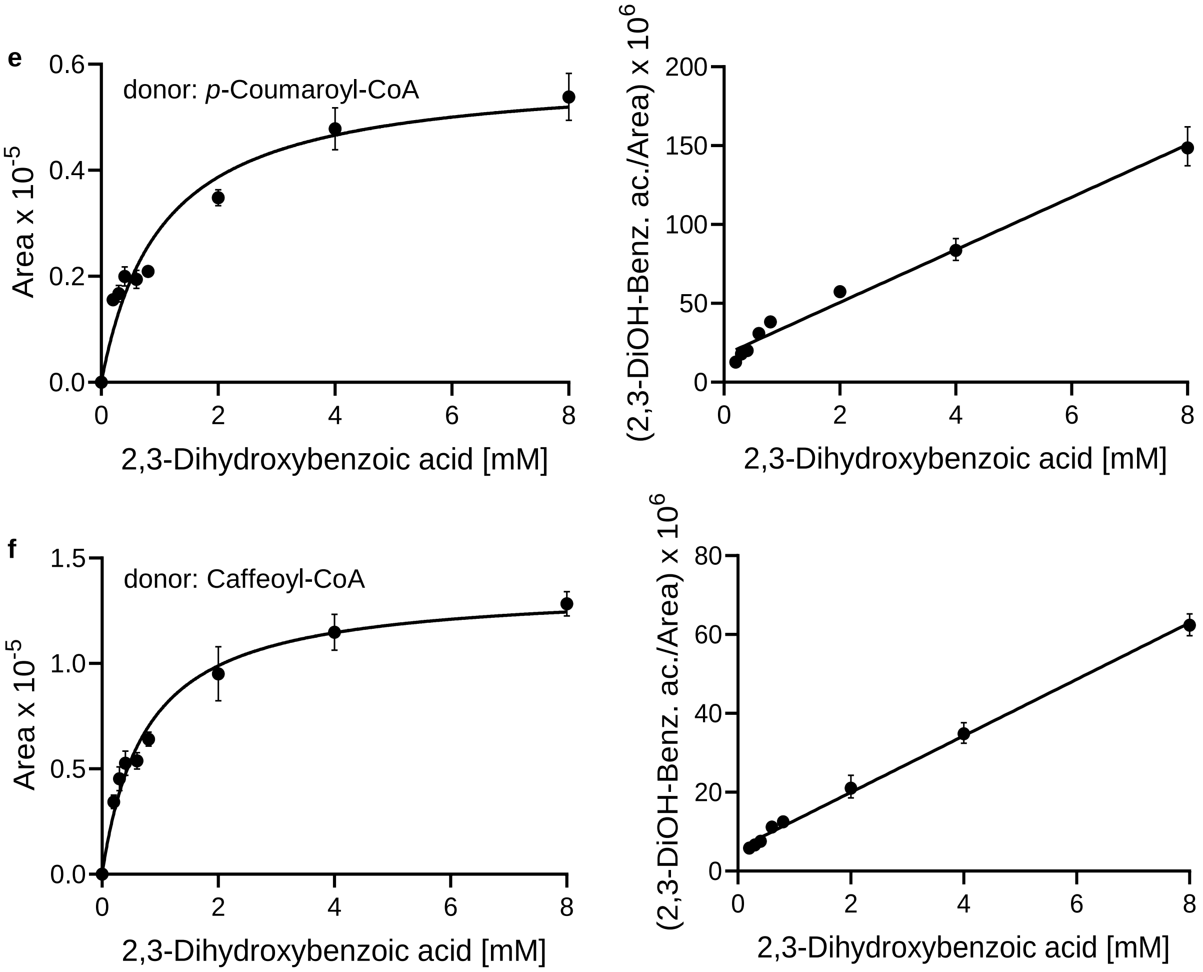

Suppl. Fig. S10 Determination of K_m-values for different acceptor substrates with *p*-coumaroyl- and caffeoyl-CoA. Michaelis-Menten (left) and Hanes-Wolf (right) diagrams for AaHCT6. **a** Shikimic acid with *p*-coumaroyl-CoA (*n* = 7 ± SE). **b** Shikimic acid with caffeoyl-CoA (*n* = 6 ± SE). **c** 3-Hydroxyanthranilic acid with *p*-coumaroyl-CoA (*n* = 12 ± SE). **d** 3-Hydroxyanthranilic acid with caffeoyl-CoA (*n* = 12 ± SE). **e** 2,3-Dihydroxybenzoic acid with *p*-coumaroyl-CoA (*n* = 3 ± SE). **f** 2,3-Dihydroxybenzoic acid with caffeoyl-CoA (*n* = 4 ± SE)

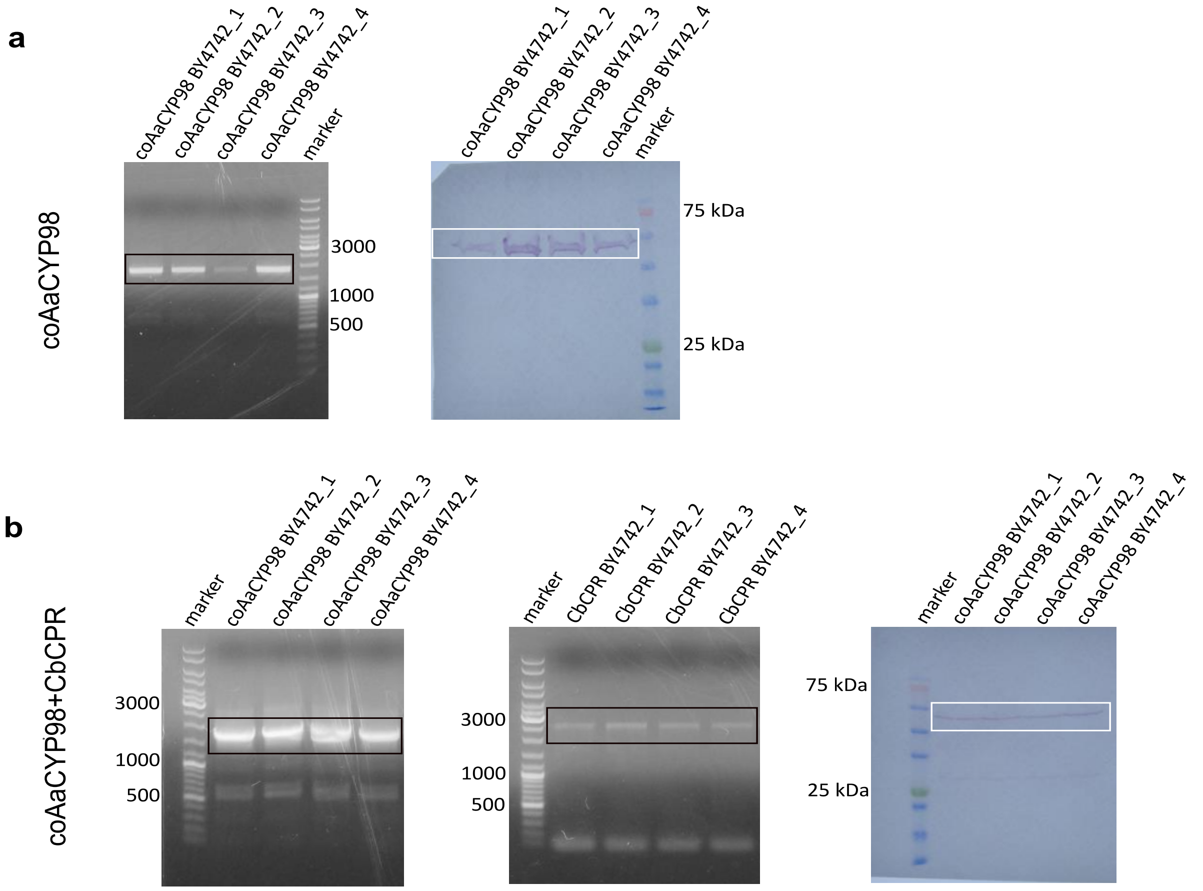

Suppl. Fig. S11 Expression of coAaCYP98 and coAaCYP98+CbCPR in *Saccharomyces cerevisiae* BY4742. **a** coAaCYP98: Colony-PCR of coAaCYP98 (left) and Western blot analysis with anti-FLAG-antibody (right). For each strain four transformants were tested. Samples (OD_{600} = 5) for Western blot analysis were taken after 48 h of incubation in SCG_{-ura} medium. **b** coAaCYP98+CbCPR: Colony-PCR of coAaCYP98 in MCSI (left) and CbCPR in MCSII (middle). Western blot analysis (right) of coAaCYP98 with anti-FLAG-antibody. Four transformants of each strain were checked and samples (OD_{600}= 5) for Western blot analysis were taken after 48 h of incubation in SCG_{-ura} medium

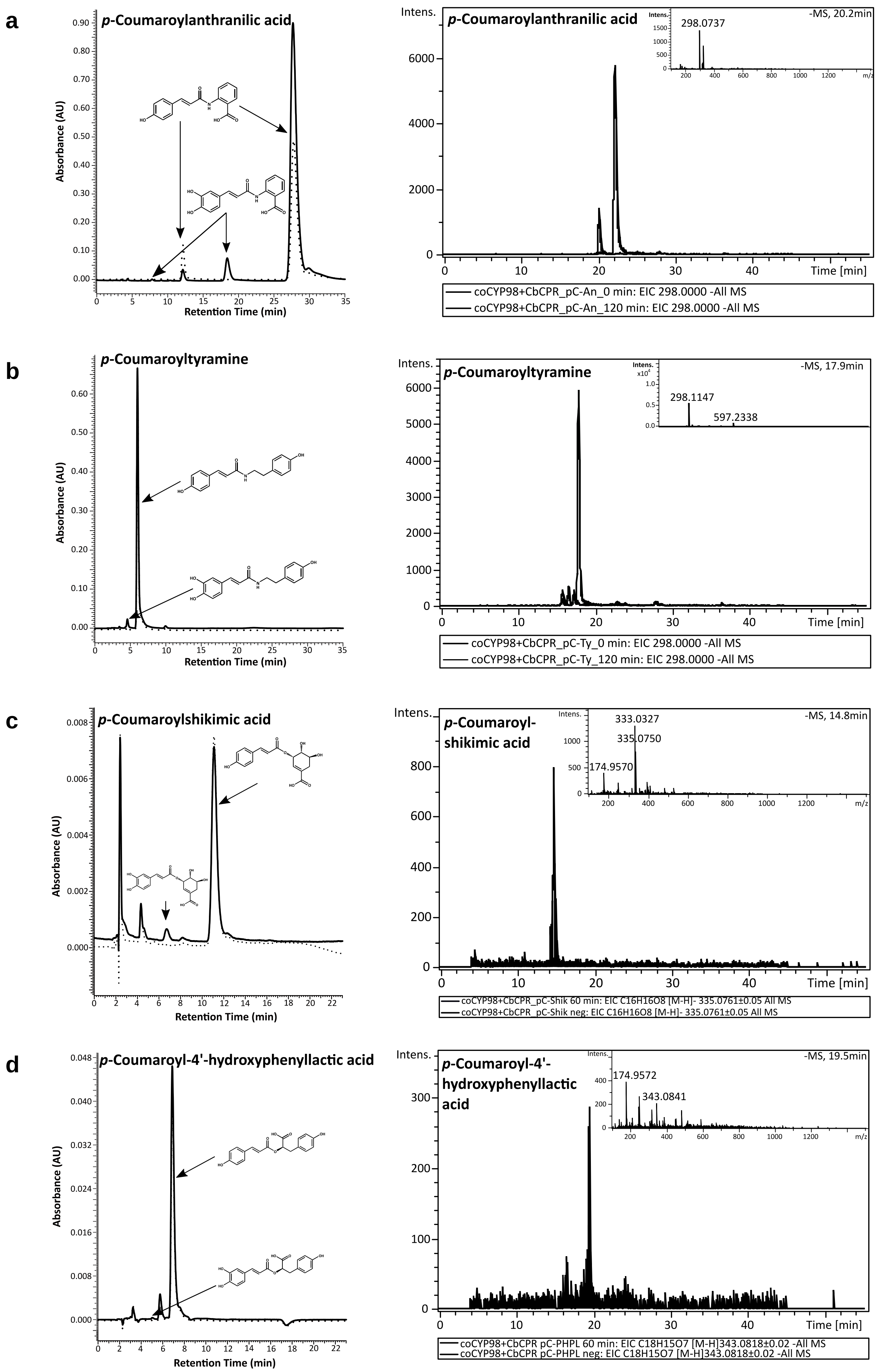
a
p-Coumaroylanthranilic acid
Absorbance (AU)
Retention Time (min)
Intens.
p-Coumaroylanthranilic acid
-MS, 20.2min
298.0737
Time [min]
coCYP98+CbCPR_pC-An_0 min: EIC 298.0000 -All MS
coCYP98+CbCPR_pC-An_120 min: EIC 298.0000 -All MS
b
p-Coumaroyltyramine
Absorbance (AU)
Retention Time (min)
Intens.
p-Coumaroyltyramine
-MS, 17.9min
298.1147
597.2338
Time [min]
coCYP98+CbCPR_pC-Ty_0 min: EIC 298.0000 -All MS
coCYP98+CbCPR_pC-Ty_120 min: EIC 298.0000 -All MS
c
p-Coumaroylshikimic acid
Absorbance (AU)
Retention Time (min)
Intens.
p-Coumaroyl-
shikimic acid
-MS, 14.8min
333.0327
335.0750
174.9570
Time [min]
coCYP98+CbCPR_pC-Shik 60 min: EIC C16H16O8 [M-H]- 335.0761±0.05 All MS
coCYP98+CbCPR_pC-Shik neg: EIC C16H16O8 [M-H]- 335.0761±0.05 All MS
d
p-Coumaroyl-4'-hydroxyphenyllactic acid
Absorbance (AU)
Retention Time (min)
Intens.
p-Coumaroyl-4'-
hydroxyphenyllactic
acid
-MS, 19.5min
174.9572
343.0841
Time [min]
coCYP98+CbCPR pC-PHPL 60 min: EIC C18H15O7 [M-H]343.0818±0.02 -All MS
coCYP98+CbCPR pC-PHPL neg: EIC C18H15O7 [M-H]343.0818±0.02 -All MS

Suppl. Fig. S12 Activity of coAaCYP98+CbCPR with different esters and amides of *p*-coumaric acid. **a** *p*-Coumaroylanthranilic acid: HPLC chromatogram (333 nm) after incubation for 0 min (dashed line) or 120 min (solid line) at 25 °C (left). Both peaks each for *p*-coumaroylanthranilic acid and caffeoylanthranilic acid are marked by arrows. Extracted ion chromatogram (EIC) and mass of caffeoylanthranilic acid obtained by LC-MS analysis (0 min grey, 120 min black) (right). **b** *p*-Coumaroyltyramine: HPLC chromatogram (333 nm) 0 min reaction time (dashed line) and 120 min (solid line) at 25 °C (left). EIC and mass of caffeoyltyramine obtained by LC-MS analysis (0 min grey, 120 min black) (right). **c** *p*-Coumaroylshikimic acid: Heat-denatured protein (5 min, 95 °C) as negative control (dashed line), assay after 60 min at 30 °C (solid line). Mass of caffeoylshikimic acid from LC-MS analysis (right: grey = negative control, black = CYP98 assay). **d** *p*-Coumaroyl-4'-hydroxy-phenyllactic acid: HPLC chromatogram at 333 nm (left) and LC-MS analysis (right). Assay incubated for 60 min at 30 °C (solid line), negative control (boiled protein, dashed line). Corresponding mass (inset) of caffeoyl-4'-hydroxyphenyllactic acid or alternatively *p*-coumaroyl-3,4-dihydroxyphenyllactic acid (grey = negative control, black = CYP98 assay after 60 min)

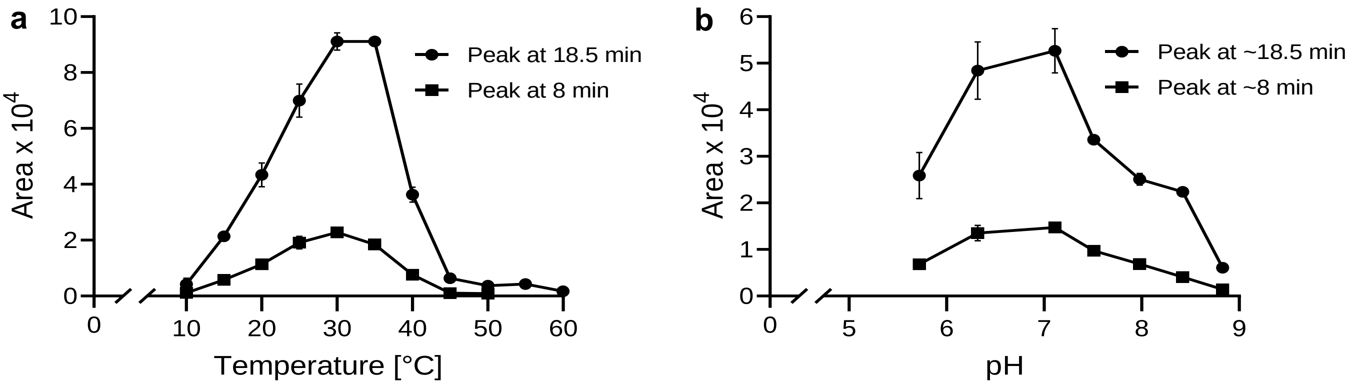

Suppl. Fig. S13 a Temperature optimum of coAaCYP98+CPR determined with *p*-coumaroyl-anthranilic acid. **b** pH-optimum of coAaCYP98+CPR determined with *p*-coumaroylanthranilic acid. Since caffeoylanthranilic acid always appeared as two peaks with identical masses but different retention times, both peaks are shown here but display the same optima for temperature and pH

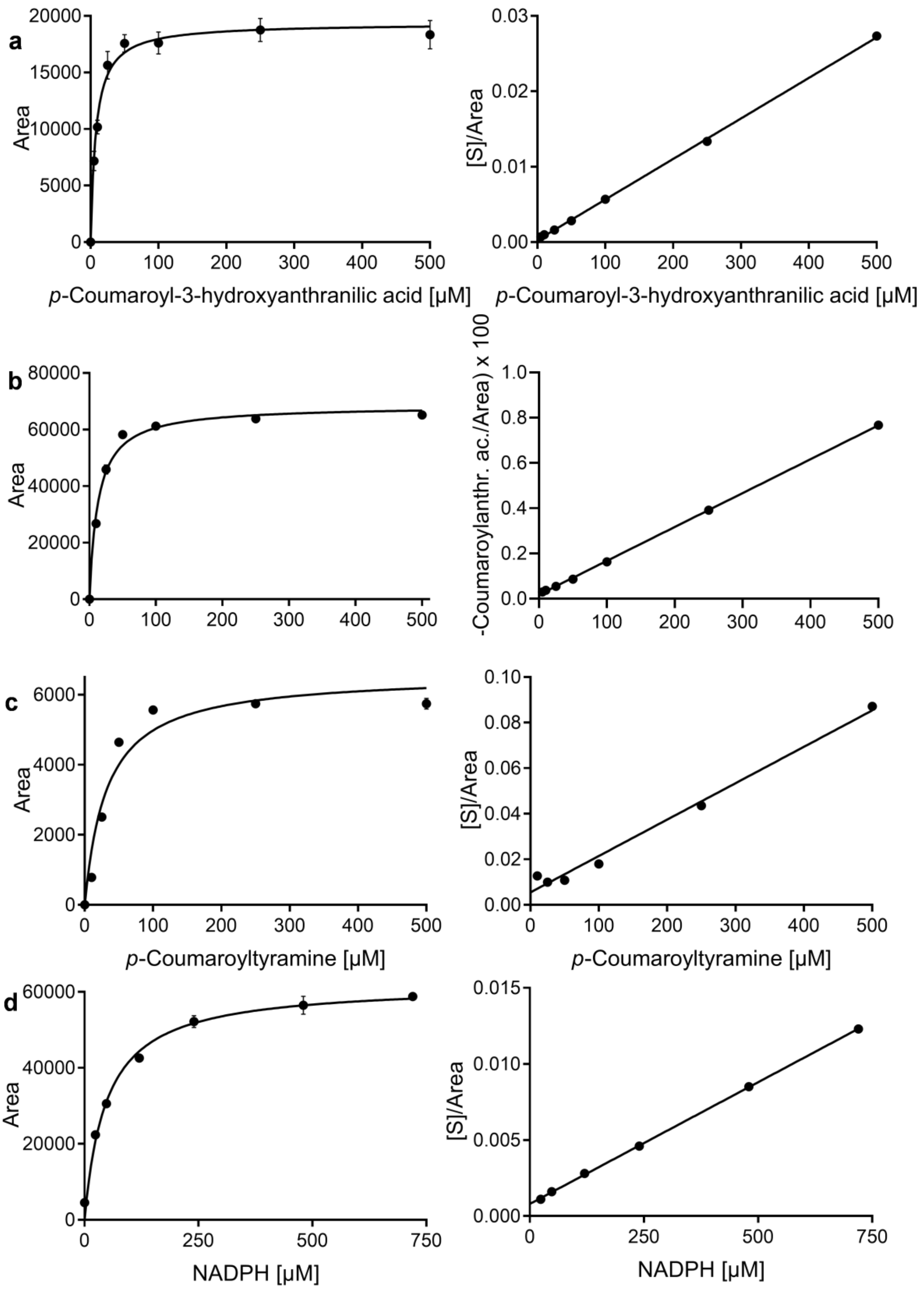

Suppl. Fig. S14 Determination of K_m-values for different substrates. Michaelis-Menten (left) and Hanes-Wolf (right) diagrams for coAaCYP98+CPR. **a** *p*-Coumaroyl-3-hydroxyanthranilic acid (*n* = 3 ± SD). **b** *p*-Coumaroylanthranilic acid (*n* = 9 ± SD). **c** *p*-Coumaroyltyramine (*n* = 3 ± SD). **d** NADPH (*n* = 3 ± SD)

Supplementary references

Jones DT, Taylor WR, Thornton JM (1992) The rapid generation of mutation data matrices from protein sequences. Comput Appl Biosci 8:275-282. doi.org/10.1093/bioinformatics/8.3.275

Kumar S, Stecher G, Tamura K (2016) MEGA7: Molecular Evolutionary Genetics Analysis version 7.0 for bigger datasets. Mol Biol Evol 33:1870-1874. doi.org/10.1093/molbev/msw054
